# Toxicity Assessment of Wild Mushrooms from the Western Ghats, India: An *in Vitro* and Sub-Acute *in Vivo* Study

**DOI:** 10.3389/fphar.2018.00090

**Published:** 2018-02-13

**Authors:** S. Sai Latha, S. Naveen, C. K. Pradeep, C. Sivaraj, M. G. Dinesh, K. R. Anilakumar

**Affiliations:** ^1^Nutrition, Biochemistry and Toxicology, Defence Food Research Laboratory (DRDO), Mysore, India; ^2^Mushroom Research Lab, Jawaharlal Nehru Tropical Botanic Garden and Research Institute, Thiruvananthapuram, India; ^3^Phytochemistry and Natural Products, Armats Biotek Training and Research Institute, Chennai, India; ^4^Herbal and Indian Medicine Research Laboratory, Sri Ramachandra University, Chennai, India

**Keywords:** mycetism, wild mushrooms, toxicity, NCM460 cell line, Chang's liver cell line

## Abstract

**Background:** Poisoning by different kinds of toxic mushrooms is unfortunately becoming an increasingly important medical problem, evident from the growing number of reports worldwide since the 1950s. Mycetism being a health concern, deserves scientific attention. In this perspective, the present study aims to assess the potential effects of ingesting the selected wild mushrooms from regions of the Western Ghats, India.

**Methods:** The preliminary cytotoxicity of the selected mushrooms was studied *in vitro* on the intestinal NCM460 and the Chang's liver cell lines on the basis of cell viability. Further, the hepatotoxicity was assessed by measuring biologically relevant endpoints such as membrane integrity, mitochondrial stress and oxidative status. A 28 day sub-acute toxicity study was carried out by orally administering the mushroom extracts to mice at 250 and 500 mg/kg body weight. The hematological and serum analysis as well as histological examinations were carried out to evaluate their *in vivo* toxicity. GC-MS analysis of the mushrooms facilitated the identification of their volatile chemical profile.

**Result:** The *in vitro* intestinal cytotoxicity exhibited by these wild mushrooms in comparison to the edible mushroom indicated their potential gastrointestinal toxicity. The pathological findings in small intestine on exposure to *Chlorophyllum molybdites* and *Agaricus endoxanthus* also validates the speculations about their intestinal toxicity. The toxic insult to the hepatocytes due to *Amanita angustilamellata, Entoloma crassum*, and *Clarkeinda trachodes* was predictive of the observed *in vivo* hepatotoxicity which was also accompanied by renal toxicity at the higher dose of 500 mg/kg bwt.

**Conclusion:** The potential toxicity exhibited by these representative mushrooms from the wild warrants caution about their consumption. The present work could also have broader implications for global mycetism.

## Introduction

By ages, foraging and eating wild mushrooms, though a favorite pastime of many, is a great health concern due to the high risk of mushroom poisoning involved in it Berger and Guss (2005). Poisoning by different kinds of toxic mushrooms is unfortunately becoming an increasingly important medical problem, evident from the growing number of reports worldwide since the 1950s (Diaz, 2005). In the global perspective, the incidence of mushroom poisoning varies according to diverse lifestyle and economic factors (Persson, 2012). The North American Mycological Association in 2014 documented 78 mushroom poisoning incidents in humans (Beug, 2014). The year 1994–2012 reported 183 deaths in China (Chen et al., 2014) and about 102 cases at Iraq, during 2006–2012 (Badsar et al., 2013) have been attributed to mushroom poisoning. Mushroom poisoning in Thailand is closely related to the lifestyle; with 38.73% of victims being agricultural workers (Saoraya and Inboriboon, 2013). It is quite obvious that mushroom poisoning is emerging as a global epidemiology (Diaz, 2005).

Of the 357 genera of Basidiomycetes (Club fungi) in the world, 232 genera have been reported in India (Manoharachary et al., 2005). Interestingly, among the 1,200 Indian species documented, only 300–315 species are considered edible (Thiribhuvanamala et al., 2011). However, mushrooms have been an integral part of the diet of many tribes and ethnic groups in India who consume nearly 283 species of wild mushrooms (Purkayastha and Chandra, 1985). As there exists no universal rule for differentiating a toxic mushroom from an edible one, these mycophilic communities, despite being guided by their traditional and indigenous knowledge in picking the wild edible mushrooms, are easily misled by their poisonous look-alikes. Thus, the monsoon months which mark the mushroom bloom, witness many cases of poisoning; a few reported and many undocumented due to the lack of confirmatory evidence (Verma et al., 2014). The spectrum of clinical manifestations due to such poisonings range from mild gastrointestinal disturbances to cytotoxicity resulting in hepatic or renal injury and even sometimes proves fatal (Gopinath et al., 2011).

**Figure 1:**
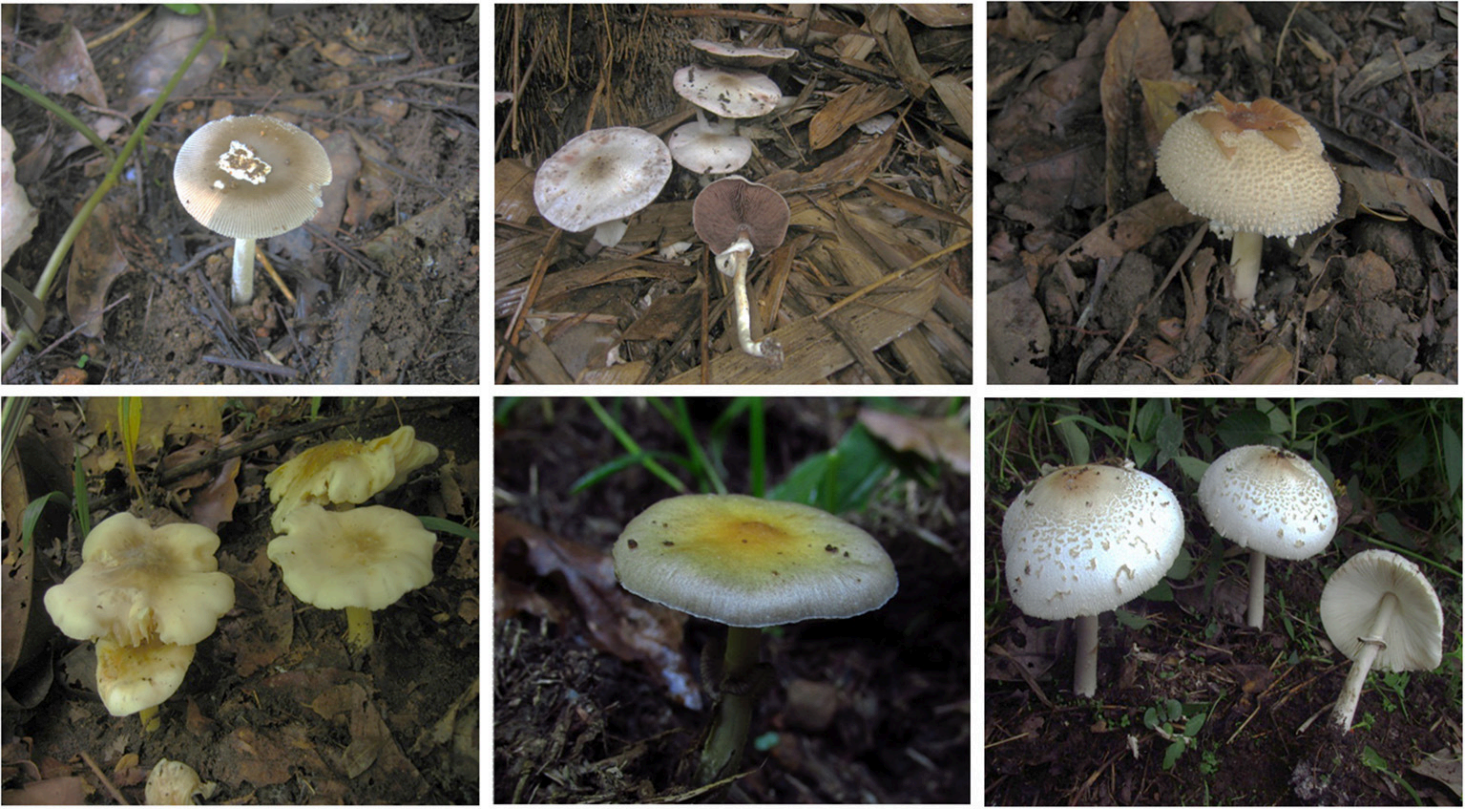
The wild mushrooms under the study (in clockwise): *Amanita angustilamellata, Agaricus endoxanthus, Clarkeinda trachodes, Chlorophyllum molybdites, Psilocybe subcubensis, Entoloma crassum*.

In the Indian subcontinent, poisonings are of common occurrence in regions where people heavily depend on wild resources for their food. In view of the growing incidence of mushroom poisonings in India, for instance the case of muscarinic poisoning recorded by George and Hegde (2013) or in the ones reported by the local newspapers such as The Kaimudi Online or The Shillong Times, it is essential to characterize the toxicological profile of the wild mushrooms before they are considered safe for consumption (Lima et al., 2012). Recently, it was also observed that the number of mushroom poisoning cases showed an upward trend in the state of Kerala, India, and in most of these cases, the main reason was found to be misidentification of the mushrooms (Bijeesh et al., 2017). The six species (Figure 1) selected for the present study are quite common and abundant in the state and fruit largely in groups as that of some edible mushrooms like *Termitomyces, Agaricus*. Their close morphological resemblance to edible mushrooms increases their probability of being picked up by amateurs and which as well tricks even the experienced into the risk of their mistaken identity (Supplementary Image 1).

The mushrooms of *Amanita* are estimated to be responsible for about 95% of the fatal mushroom poisonings worldwide and thus have been subjected to careful scrutiny (Vetter, 1998). The saprobic genus *Agaricus* comprises not only the edible species but also potentially toxic ones, especially those belonging to the section *Xanthodermatei* (Thongklang et al., 2014). Therefore, this study has considered evaluating the toxicity of *Amanita angustilamellata* and *Agaricus endoxanthus* which represent the speculated sections of *Amanita* and *Xanthodermatei*, respectively. Since 1900, the medical and botanical literature has been citing the poisonings associated with *Chlorophyllum Molybdites* (Bijeesh et al., 2017) and it is one of the most easily misidentified species (Cochran, 1987). However, the edibility of this mushroom has also been discussed controversially as having been associated with serious gastrointestinal troubles (Pegler and Piearce, 1980) while contradicted by Guissou et al. (2008) as harmlessly edible. The toxic symptoms of this mushroom are seemingly debatable due to the variations on account of geographical distribution, personal idiosyncrasies associated with its consumption and also the effectiveness of cooking processes involved in its preparation (Young, 1989). Therefore, as a part of our study, an attempt was made to verify the toxicity of this mushroom.

The literature on the taxon *Entoloma* has also been inconsistent regarding its edibility aspects. For example, *E. clypeatum* is good and edible according to Svrček, 1988, while considered suspicious by Phillips (2006) and in some cases found to be toxic when eaten raw (Bresinsky, 1990). The poisonings associated with *E. sinuatum* (Hanrahan and Gordon, 1984) and *E. niphoides* (Becker, 1960) have reiterated the point made by Pegler and Watling (1982), that the gray and brown species of *Entolomataceae* are best avoided. *Entoloma* species are abundant in the state of Kerala (Manimohan et al., 2006) and the new species *Entoloma crassum* shares many microscopic and macroscopic similarities with its toxic relatives *E. sinnuatum* and *E. niphoides* (Pradeep et al., 2012) and hence this study explores its toxic potential. *Psilocybe* mushrooms referred to as “magic mushrooms,” are usually associated with psychiatric complications and are considered as one of the major natural hallucinogenic drugs of abuse (van Amsterdam et al., 2011). *Psilocybe subcubensis* is considered as the pantropical sister species of *Psilocybe cubensis* which is known to cause psychedelic effects. This *Psilocybe* species is an underexplored representative of its genus and hence has been included in the current study. To the best of our knowledge, this study is the first report about the toxicity of *Clarkeinda trachodes*, which has been mentioned as poisonous, but without any substantial scientific evidence. Also as the genera of these mushroom species are speculated to be toxic according to the POISINDEX Information System (Rumack and Spoerke, 1993; Supplementary Table 1), the present work aims to provide a scientific validation of the possible outcomes of consuming these mushrooms. So far, there are no preceding reports of research on the edibility aspects of the selected mushrooms.

The present study has been designed to evaluate the potential effects on ingestion of the selected wild mushrooms and thereby in order to ascertain the edibility or toxicity of these indigenous species. The *in vitro* cytotoxic potential of the selected mushrooms was assessed using two cell line models, the intestinal and the liver. The assessment was conducted by measuring biologically relevant endpoints such as the cell viability, membrane integrity, mitochondrial stress, and oxidative status. Further, the oral toxicity study was carried out in mice to identify the *in vivo* toxic responses on administering the mushroom extracts at two doses which correspond to about half and one serving of mushrooms in case of humans. Hematology analysis, serum biochemical assays and histological examinations were performed in order to evaluate toxicological profile of the mushrooms. Also, the GC-MS analysis facilitated the identification of characteristic volatile profile of these mushrooms. The findings of this work may serve as significant cues in deciding the edibility of these mushrooms.

## Materials and methods

### Chemicals and kits

The chemicals used for the study, Minimum Essential Medium (MEM), penicillin-streptomycin solution, MTT (3-[4, 5-dimethyl thiazol-2-yl]-2, 5-diphenyl-2-H- tetrazolium bromide), DCFDA (2′, 7′-Dichloro dihydrofluorescein diacetate), Rhodamine 123, Griess reagent were purchased from Sigma Aldrich (India). Fetal bovine serum (FBS) was bought from HyClone™ (USA). The kits used for the estimation of Lactate dehydrogenase (LDH) and the serum biochemistry parameters were purchased from Agappe Diagnostics Ltd., India.

### Collection of mushroom samples

The mushroom samples were collected from different forest localities of Wayanad and Trivandrum districts of Kerala state, India, during the period of June–August, 2013.The collected mushroom specimens were systematically studied for their macroscopic as well as microscopic characteristics and identified using standard taxonomic keys (Singer, 1986). All the specimens studied were deposited in the Mycological Herbarium of Jawaharlal Nehru Tropical Botanic Garden and Research Institute, Trivandrum [TBGT]. The six wild mushrooms under this study along with their voucher numbers are as follows: *A. angustilamellata* (AL) (TBGT11861), *P. subcubensis* (PS) (TBGT12004), *A. endoxanthus* (AE) (TBGT14768), *E. crassum* (EC) (TBGT145817), *C. trachodes* (CT) (TBGT14500), *Chlorophyllum molybdites* (CM) (TBGT14944), and *Agaricus bisporus* was purchased from the local market, Mysore.

### Preparation of the extract

The specimens collected were shade dried and stored under controlled temperature of 10°C. The mushrooms were then coarsely powdered and extracted with 50% ethanol. About 100 mL of the solvent was used for 5 g dry weight of the mushrooms and extraction was carried out overnight on continuous shaking. This extraction process was repeated and the filtrates from each extraction were pooled and subjected to rotary evaporation in vacuum to remove the alcohol. Dry powder of the extracts was obtained after subjecting the alcohol free extracts to lyophilization. The extracts thus obtained were tested for their toxic effects *in vitro* and *in vivo*. The *in vitro* and *in vivo* response to the selected mushroom extracts was compared with the identically prepared extract of the nontoxic mushroom *A. bisporus* (Prast et al., 1988).

### Cytotoxicity evaluation

#### Cell lines

The NCM460 (colon epithelial cells) and Chang's liver cell lines were obtained from National Centre for Cell Sciences (NCCS), Pune, India, and grown at 37°C in a humidified atmosphere of 5% CO_2_ using MEM, supplemented with 10% FBS and antibiotic solution. About 80% confluent cells were used for the assays which were carried out in triplicates.

#### Cell viability assay

The MTT assay was used to assess the *in vitro* cytotoxicity of the mushroom extracts on NCM460 and Chang's liver cell lines. The cells (100 μL; 1 × 10^5^ cells/mL) were seeded into 96 well microtiter plates and left to adhere for 24 h. The medium was then removed from the wells and replaced with 100 μL filter sterilized medium containing the mushroom extracts. The cells were incubated with different concentrations (0–2.5 mg/ml) of the extracts for the next 24 h. MTT (100 μl; 0.5 mg/mL) was added to each well and further incubated for 2 h. The medium was removed and DMSO (100 μL) was added. The absorbance was read at 570 nm (Mosmann, 1983) and the cell viability was expressed as percent of control. The toxic effects of the extracts on cell morphology were also noted and photographed. On completion of the preliminary viability assay with both the cell lines, the further assays were carried out using the Chang's liver cell line.

#### Lactate dehydrogenase leakage assay

The LDH estimation kit (Agappe Diagnostics Ltd.) was used to measure the extent of plasma membrane damage. The cells (100 μL; 1 × 10^5^ cells/mL) were seeded into 96 well plates and left for about 24 h to adhere. The cells were exposed to the mushroom extracts (100 μL; 1 mg/mL) for the next 24 h and the amount of LDH leaked into the medium was estimated at 340 nm in terms of the reduced nicotinamide adenine dinucleotide (NADH) formed.

#### Estimation of nitric oxide levels

The Griess reagent was used to estimate the nitric oxide levels in terms of the amount of nitrite generated. After 24 h exposure of the cells to the mushroom extracts, to the culture supernatant equal amount of the Griess reagent was added and allowed to react for 5 min at room temperature. The absorbance was measured at 540 nm (Banati et al., 1993).

#### Estimation of intracellular oxidative stress

The oxidative stress in terms of the reactive oxygen species (ROS) generated was measured using the dye DCFDA following the procedure of Wang and Joseph (1999). The cells seeded in 24 well plates were exposed to the mushroom extracts as mentioned earlier. After 24 h of exposure, the oxidation sensitive dye DCFDA (10 μg/mL) was added and incubated for an hour. The cells were washed thrice with PBS and the intracellular ROS formation was detected at an excitation wavelength of 485 nm and an emission wavelength of 535 nm using microplate reader (Hidex plate chameleon™V, Finland).

#### Assessment of mitochondrial membrane potential

A modified protocol of Rahn et al. (1991) was used to assess the effect of the mushroom extracts on the mitochondrial membrane potential using the fluorescent dye Rhodamine 123. After exposing the cells to the extracts for 24 h, 100 μL of Rhodamine 123 (10 μg/mL) was added to the wells and incubated for 1 h at 37°C. The cells were then washed thrice with PBS and the fluorescence intensity was measured at excitation wavelength of 485 nm and an emission wavelength of 535 nm using microplate reader (Hidex plate chameleon™V, Finland).

### Sub-acute oral toxicity study in mice

#### Animals and housing conditions

Female Balb/C mice of body weight between 25 and 30 g, from the Central Animal Facility of the laboratory were used for the study. Six animals per cage were housed in standard sized cages with sawdust bedding and maintained under controlled 12 h light/dark cycle, optimum conditions of temperature and relative humidity (23 ± 2°C, 40–60% humidity). Potable water and standardized pellet diet (Diet composition as per Supplementary Table 2) were provided *ad libitum* to the animals. The experimental procedures were performed in accordance with the Ethical Principles in Animal Research and approved by the Ethics Committee, Defense Food Research Laboratory, DRDO (Ref no. DFRL/IAEC/01/2015). The study design followed the OECD guidelines TG 407 (OECD, 2008) for assessing the sub-acute toxicity.

#### Dose formulation and administration

The mice were randomly divided into 15 groups (*n* = 6). The animals were fasted for 2 h prior to the administration of the mushroom extracts *A. angustilamellata (AL), P. subcubensis (PS), A. endoxanthus (AE), E. crassum (EC), C. trachodes (CT), C. molybdites (CM)*, and *A. bisporus (AB)* and the control animals were administered water in place of the extracts. The mushroom extracts were dissolved in water and each mushroom extract was administered at 250 and 500 mg/kg bwt every day for 28 consecutive days. On administration of the extracts, the animals were observed for signs of toxicity and mortality during the study period. At the end of the study, the animals were sacrificed under anesthetic conditions and blood was drawn through cardiac puncture. The body weights were measured on the day 14 as well as prior to sacrifice of the animals.

#### Hematological analysis

For hematological analysis, the red blood cell count (RBC), hemoglobin concentration (HGB), hematocrit (HCT), platelets (PLT), white blood cell count (WBC), and lymphocytes (LYM) were measured in the uncoagulated blood using an automated hematology analyzer, Sysmex KX-21 (Tranasia Bio-medicals Pvt. Ltd., India).

#### Blood biochemical analysis

Biochemical parameters in serum were analyzed to assess the liver and kidney functioning using the commercially available kits from Agappe. The liver function tests (LFTs) included the estimation of enzyme activities such as aspartate transaminase (AST), alanine aminotransferase (ALT), alkaline phosphatase (ALK), and the total bilirubin (TBIL) content. The kidney function tests (KFTs) included the estimation of creatinine (CRE), blood urea nitrogen (BUN), and total protein (TPR).

#### Histopathological studies

The organs viz., brain, kidney, liver, small, and large intestine were excised on sacrificing the animals and prepared for histopathological analysis by embedding in paraffin wax and staining with hematoxylin and eosin.

### GC-MS analysis

GC-MS analysis was performed with Agilent GC 6890 N JEOL GC Mate II with Ion Trap gas-chromatograph equipped with HP5 capillary column (30 m × 0.32 mm; coating thickness 0.25 μm) interfaced with Agilent 240 MS Ion Trap mass detector. Analytical conditions were as follows: Injector and transfer line temperature 20 and 250°C, respectively; oven temperature programmed from 50 to 250°C at 10°C/min; helium as the carrier gas at 1 mL/min; ionization voltage of 70 eV; ion source temperature of 250°C; interface temperature of 250°C; mass range of 50–600 mass units. The identification of the components was performed for both the columns by comparison of their linear retention indices and by computer matching against commercial mass spectra libraries NIST and MS literature data.

### Statistical analysis

The results were represented as the mean ± *SD* and the statistical significance was analyzed using one-way analysis of variance (ANOVA) followed by Tukey's HSD-*post-hoc* test. All statements of differences were based on significance at *p* < 0.05. The statistical analyses and graphical representations were carried out using Graph Pad Prism software version 5.

## Results

The mushroom specimens studied as a part of this work (Figure 1), were sampled during the monsoon months from Trivandrum and Wayanad districts of Kerala and identified up to the species level using the standard taxonomic keys. The yield on extraction was about 3.5% of the fresh weight of the mushrooms and the extracts thus obtained were used for the toxicity studies *in vitro* and *in vivo*.

### *In vitro* toxicity

#### Cell viability

**Figure 2:**
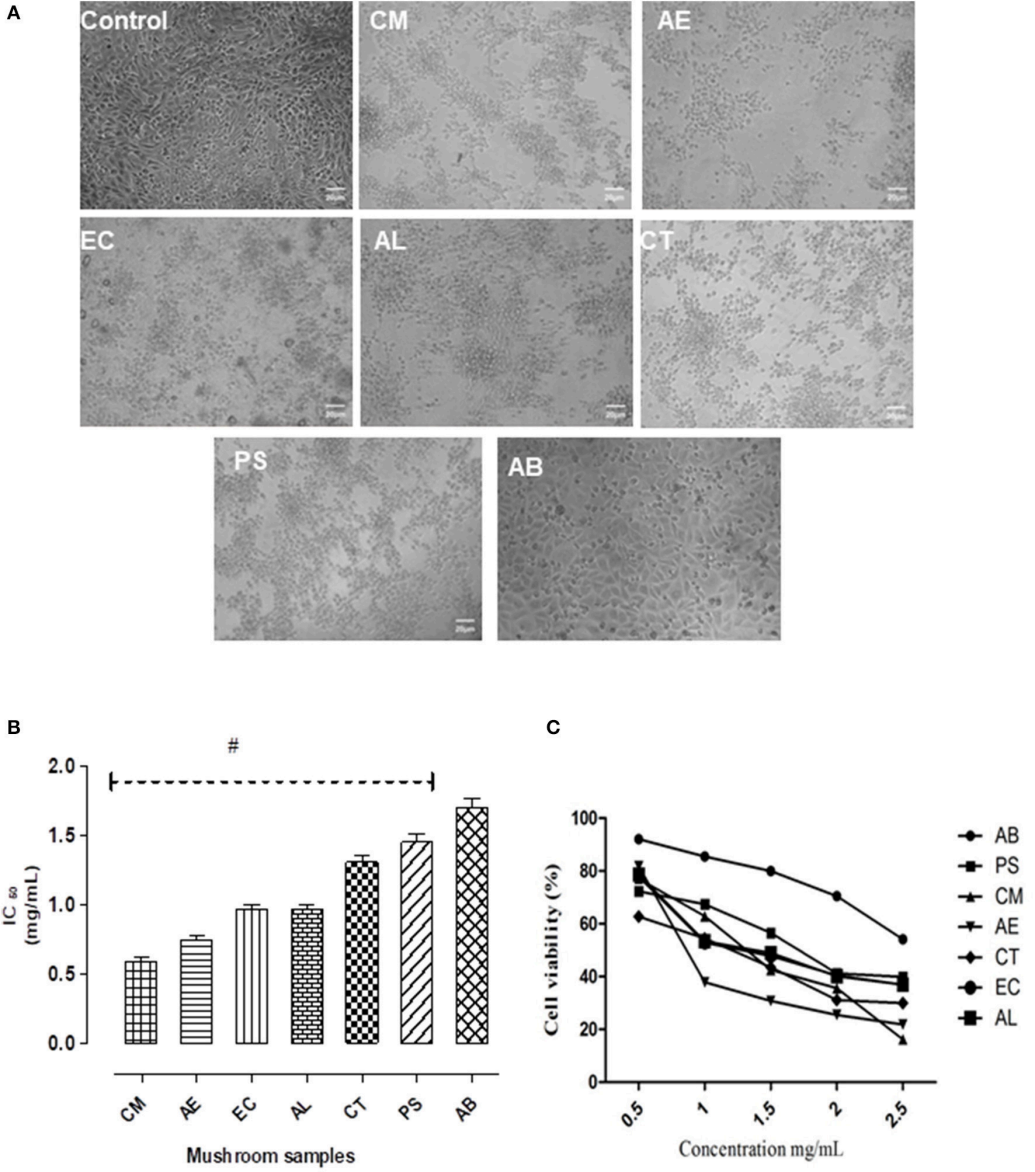
**(A)** The effect of the extracts on the morphology of NCM460 cells as visualized under a phase contrast microscope. **(B)** IC_50_ values of the extracts. **(C)** Dose response curves w.r.t. exposure to different concentrations of the mushroom extracts on the intestinal cells. Data presented are the means ± *SD* of results from three independent experiments. “#” represents significant difference with respect to AB at *p* < 0.05.

On 24 h exposure to the mushroom extracts, there were distinct changes in the morphological structure of the cells (Figure 2A). The cytotoxic effects on the intestinal cells were severe in case of the mushroom CM, followed by AE and EC. However, the other mushrooms AL, CT, and PS exhibited a moderate to mild toxicity. The edible AB caused only slight structural changes in comparison to the control. The toxicity was also reflected in the IC_50_ values of the extracts with CM showing the least (0.56 mg/mL) and AB the highest (1.65 mg/mL) as shown in Figure 2B. The cell viability showed a significant (*p* < 0.05) reduction in case of all the mushroom extracts with respect to the control as well as the edible mushroom AB.

**Figure 3:**
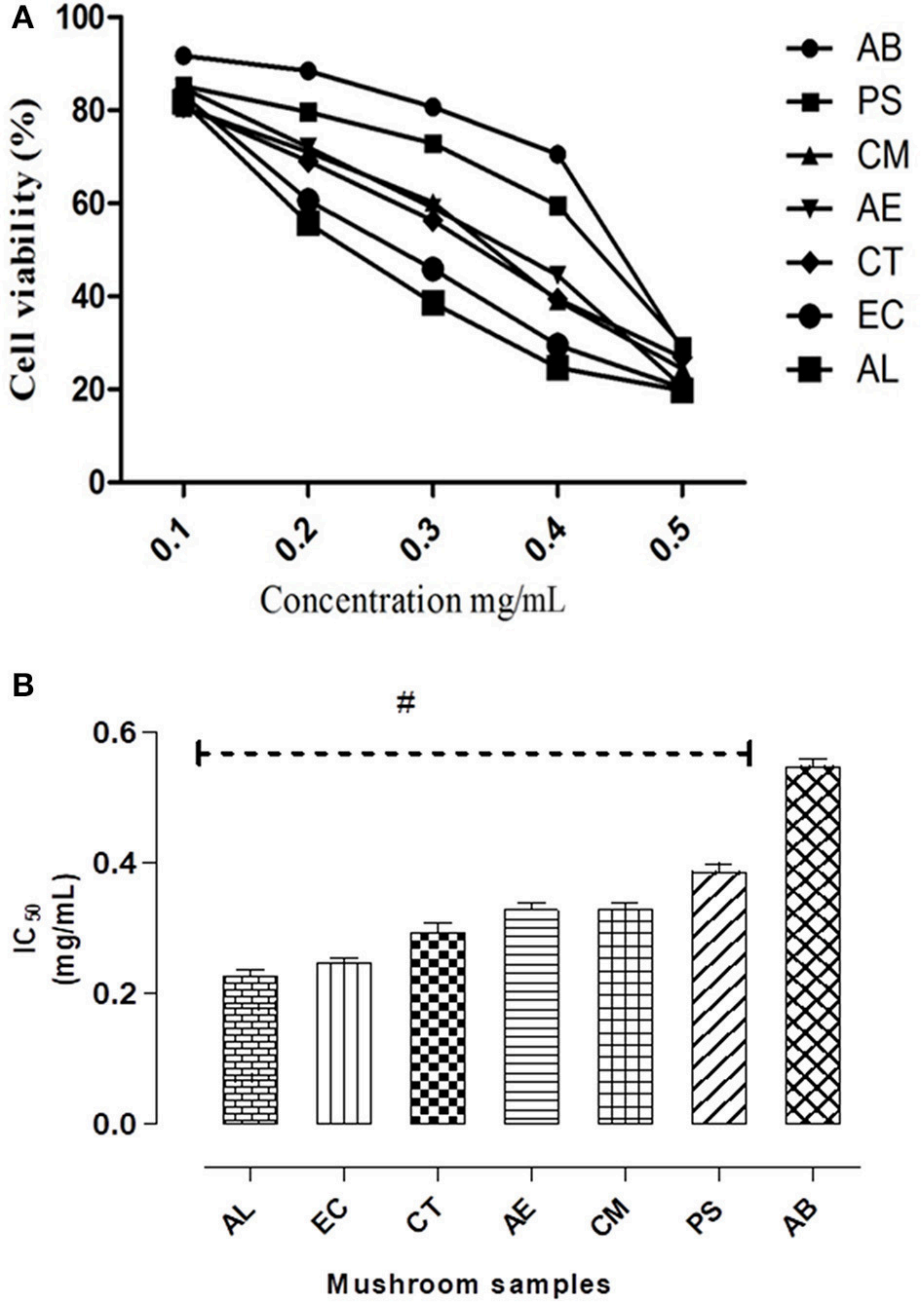
**(A)** IC_50_ values of the extracts on Chang's liver cell line. **(B)** Dose response curves w.r.t. exposure to different concentrations of the mushroom extracts on the hepatocytes. Data presented are the means ± *SD* of results from three independent experiments. “#” represents significant difference with respect to AB at *p* < 0.05.

In case of hepatocytes also, a similar dose dependent decrease in cell survival was observed on exposure to different concentrations of the extracts (Figure 3A) as was seen in the intestinal cells (Figure 2C). Their relative cytotoxicity was represented as corresponding IC_50_ values (Figure 3B). The results indicated the potential hepatotoxicity of these mushrooms. Among the mushrooms studied, AL was found to be the most toxic to the hepatocytes indicated by its significantly low IC_50_ value (0.21 mg/mL).

#### LDH leakage

**Figure 4:**
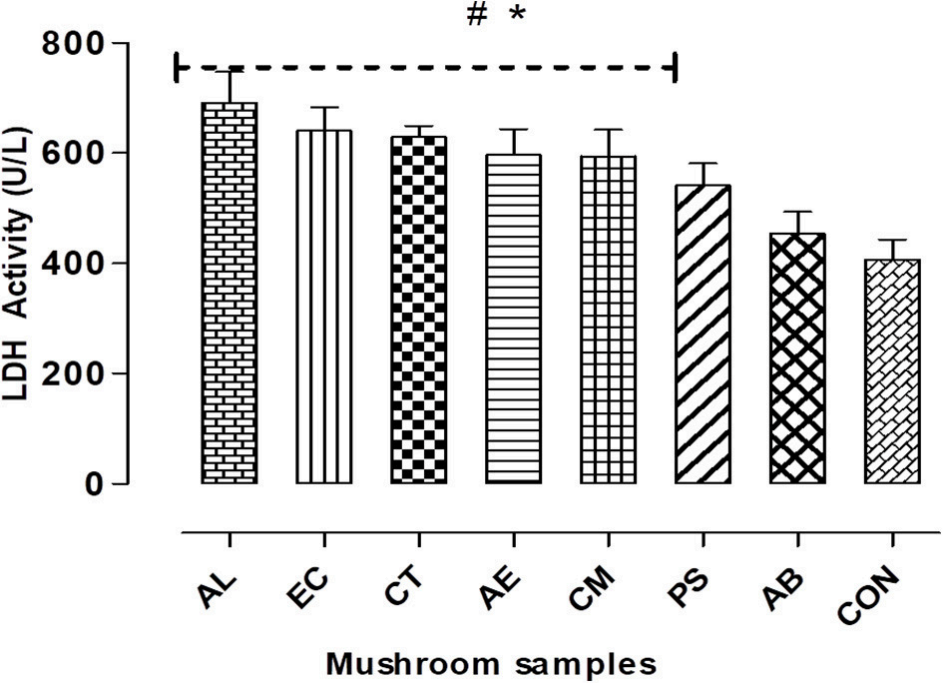
Cell membrane integrity was measured by LDH leakage assay. Data presented are the means ± *SD* of results from three independent experiments. “#” and “^*^” represents significant difference with respect to AB and control respectively at *p* < 0.05.

On assessing the membrane integrity of the hepatocytes in terms of LDH leakage, it was observed that there was a significant (*p* < 0.05) increase, about 30–70% higher than the control in case of the mushroom extracts except for AB (Figure 4). Also, it was noted that there was a relationship between the estimated extracellular LDH on exposure to the mushroom extracts and their respective IC_50_ values. The highest increase was observed in case of AL which showed the least IC_50_ and this suggests its greater potential in disrupting the plasma membrane integrity in comparison to the other mushrooms.

#### Oxidative stress

**Figure 5:**
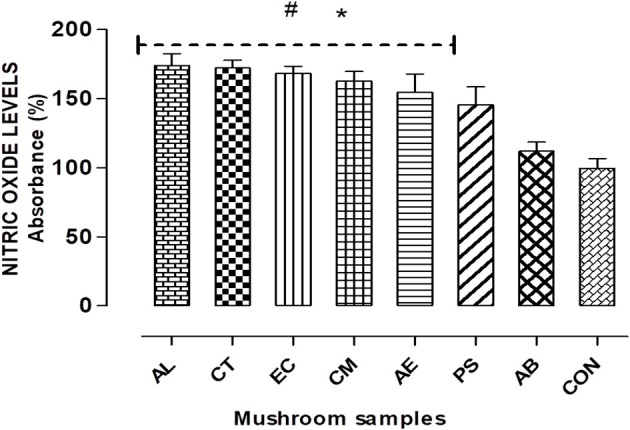
Nitric Oxide (NO) radicals generated on exposure to the extracts for 24 h. Histograms represent % increase in comparison to the control. Data presented are the means ± *SD* of results from three independent experiments. “#” and “^*^” represents significant difference with respect to AB and control respectively at *p* < 0.05.

To verify the role of oxidative stress in the cell death observed, the ROS production and nitrite levels were measured on 24 h exposure to the extracts. Figure 5 shows that the extracts elicited a significant (*p* < 0.05) elevation of about 45–75% in the NO levels compared to the control, with the highest exhibited by AL.

**Figure 6:**
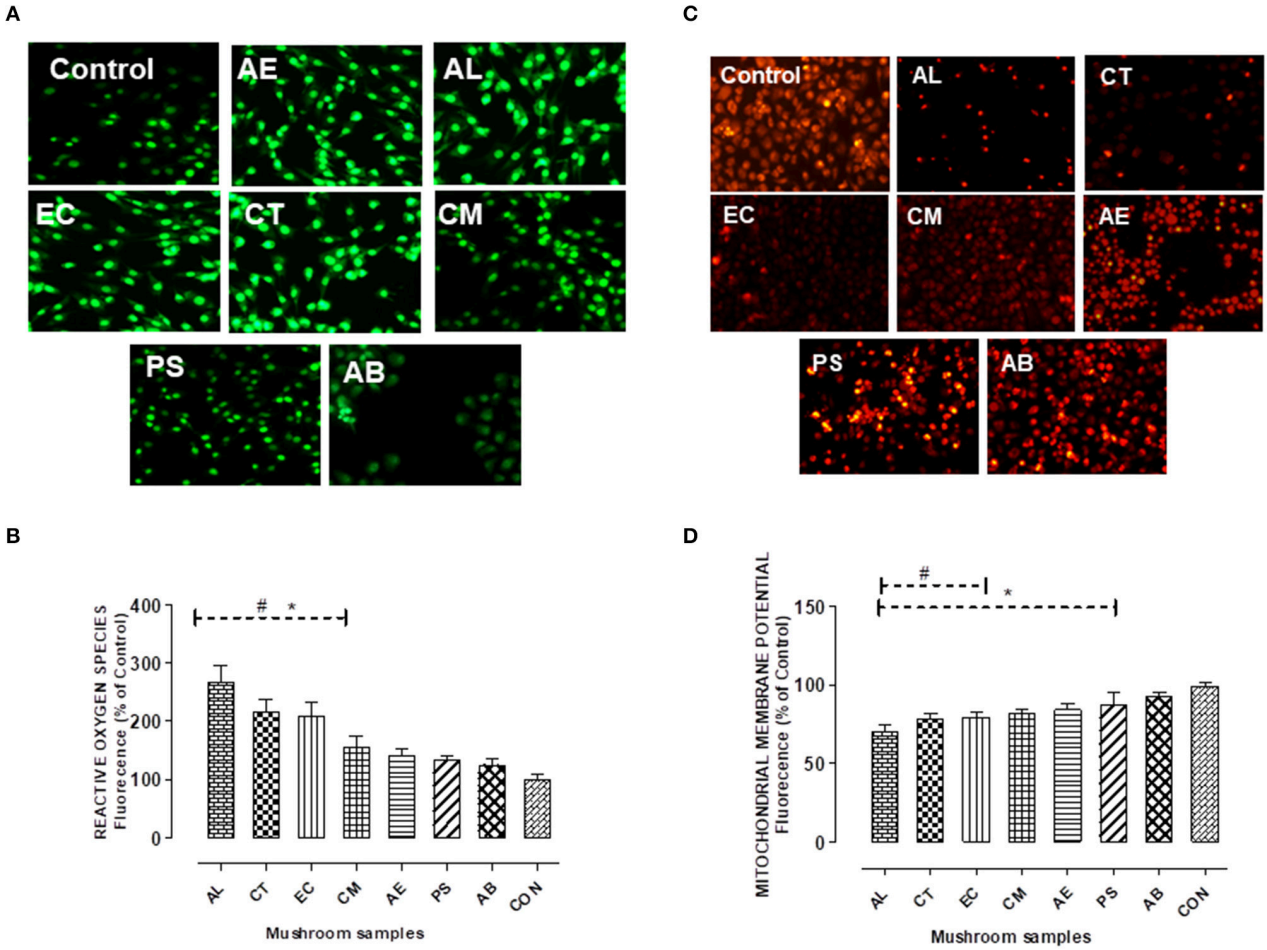
**(A,B)** Detection of intracellular reactive oxygen species using the fluorescent probe H_2_DCFDA. Chang's Liver cells were exposed to the extracts for 24 h as indicated and the fluorescence levels in the attached cells was measured at 485 nm for excitation and 515 nm for emission. **(C,D)** Mitochondrial membrane potential changes were measured by the uptake of the cationic fluorescent dye Rhodamine 123. Histograms represent % increase (in case of ROS) and decrease (in case of MMP) in comparison to the control. Data presented are the means ± *SD* of results from three independent experiments. “#” and “^*^” represents significant difference with respect to AB and control respectively at *p* < 0.05.

The cells exposed to the extracts also showed a significant (*p* < 0.05) increase in ROS generation which was observed qualitatively (Figure 6A) as well as quantified using the DCF-DA dye. The quantification of ROS generated which is proportional to the fluorescence intensity was found maximum on exposure to AL (about two and half times in comparison to control) as indicated in Figure 6B. The effect of oxidative stress on cell viability was evident as the three mushrooms AL, CT, and EC which showed significantly higher levels of ROS resulted in greater extent of cell death in comparison to the other mushrooms.

#### Mitochondrial membrane potential (ΔΨ_m_) analysis

As presented in the Figures 6C,D, the mushrooms except PS showed a significant decrease in the mitochondrial membrane potential. About 16–30% decrease in comparison to the control cells was observed on exposure to the extracts, with AL showing the maximum reduction. This was indicative of the mitochondrial stress caused by the extracts. However, there was no significant reduction in the membrane potential in case of AB in comparison to control.

### *In vivo* toxicity

#### Mortality and general observations

The oral administration of the mushroom extracts at both doses showed no mortality during the study period. However, signs of behavioral abnormalities were noticed in the animals which were administered the PS extract. These animals showed a marked decrease in their mobility in comparison to the control animals and displayed a crouching position for a few minutes after administration of the extract. The animals also exhibited a transient gnawing and grooming behavior. However, the administration of the extracts except for AB and PS caused a body weight reduction in the animals (Supplementary Image 2).

#### Effect on hematological parameters

There were variations in the hematological parameters observed on exposure to the mushroom extracts as presented in the Table 1. However, a significant (*p* < 0.05) increase was observed only with respect to the total WBC count and the LYM count at the end of the study period, except for AB. This increase also followed a dose dependent pattern in case of all the mushroom extracts. The highest WBC count was noted for the mushroom EC, which ranged between 6.59 and 9.98 (×10^3^/μL). The mushroom AL at 500 mg/kg bwt caused an elevation of the LYM to about 9.80 (×10^3^/μL) which was noted as the highest among the mushrooms. The other parameters such as PLTs, RBCs, HGB, and HCT also showed variations but were not significantly different from the control values. But within the groups such as AL500 platelet counts were significantly different between 14 and 28 days values. Also within the control groups for 14 and 28 days, there was no significant difference at *p* < 0.05 level except for LYM values. In brief, all the values were within the normal book values.

**Table 1:** Effect of the mushroom extracts on hematological parameters on day 14 and 28.

		**WBC (10^3^/μL)**	**LYM (10^3^/μL)**	**PLT (10^5^/μL)**	**RBC (10^6^/μL)**	**HGB(g/dL)**	**HCT (%)**
CONTROL	D 28	3.22 ± 0.58	1.22 ± 0.14	991.50 ± 351.93	7.89 ± 0.79	12.31 ± 1.17	40.09 ± 3.96
	D 14	2.92 ± 0.88	2.01 ± 0.47	1039.72 ± 257.89	8.62 ± 1.43	13.16 ± 1.28	38.45 ± 4.51
AB 500	D 28	4.56 ± 0.98	1.89 ± 0.36	1218.40 ± 174.06	8.45 ± 1.91	13.80 ± 1.55	44.74 ± 2.08
	D 14	4.63 ± 0.94	1.20 ± 0.13	1236.33 ± 125.69	8.87 ± 0.06	12.70 ± 0.40	45.40 ± 1.21
AB 250	D 28	5.88 ± 1.74	1.45 ± 0.44	1259.50 ± 144.24	9.99 ± 1.08	14.55 ± 1.03	51.30 ± 5.11
	D 14	3.40 ± 0.88	1.30 ± 0.56	1601.66 ± 152.26	10.61 ± 1.49	15.02 ± 1.24	52.70 ± 6.48
AE 500	D 28	5.56 ± 0.32^*^	5.46 ± 1.06^*^	662.67 ± 413.41	8.97 ± 0.62	12.46 ± 0.66	45.63 ± 3.88
	D 14	4.41 ± 0.35	3.23 ± 1.60	1083.40 ± 248.59	9.26 ± 0.27	13.50 ± 0.89	47.64 ± 2.04
AE 250	D 28	4.63 ± 0.44^*^	3.56 ± 0.84^*^	1028.50 ± 224.65	8.97 ± 0.63	13.80 ± 0.89	47.97 ± 2.76
	D 14	2.90 ± 1.23	2.40 ± 1.11	1029.00 ± 84.19	9.27 ± 0.31	13.63 ± 0.20	46.26 ± 1.34
AL 500	D 28	6.93 ± 1.33^*^	9.80 ± 0.08^*^	1148 ± 394.93	10.32 ± 1.23	14.90 ± 1.06	50.80 ± 4.44
	D 14	5.96 ± 1.52	3.28 ± 1.67	392 ± 123	4.99 ± 0.79	9.85 ± 1.59	29.9 ± 4.69
AL 250	D 28	5.50 ± 0.89^*^	5.20 ± 0.08^*^	996 ± 185.42	7.84 ± 0.79	12.29 ± 1.32	44.80 ± 3.91
	D 14	3.09 ± 0.12	2.20 ± 0.62	1381 ± 12.65	9.22 ± 0.58	14.76 ± 0.83	59.03 ± 3.09
CM 500	D 28	5.32 ± 0.88^*^	5.38 ± 0.65^*^	589.5 ± 234.77	6.96 ± 2.78	12.20 ± 0.88	37.31 ± 2.75
	D 14	1.94 ± 0.62	1.08 ± 0.25	1092.5 ± 445.31	6.91 ± 2.58	10.27 ± 3.33	37.00 ± 5.85
CM 250	D 28	5.16 ± 0.56^*^	4.07 ± 1.08^*^	593.33 ± 319.42	5.96 ± 3.17	10.56 ± 3.02	35.06 ± 5.57
	D 14	5.90 ± 2.93	1.40 ± 0.04	953.50 ± 28.98	9.06 ± 0.76	12.70 ± 0.65	44.80 ± 3.26
CT 500	D 28	5.23 ± 0.66^*^	3.67 ± 0.20^*^	1403.66 ± 26.88	8.75 ± 0.53	13.63 ± 1.41	46.90 ± 2.73
	D 14	2.63 ± 0.62	2.50 ± 0.87	988.50 ± 27.94	9.12 ± 0.28	14.14 ± 0.96	47.96 ± 2.83
CT 250	D 28	4.45 ± 0.20^*^	2.20 ± 0.06^*^	1121.33 ± 106.42	8.47 ± 0.60	12.66 ± 0.78	42.23 ± 2.84
	D 14	2.40 ± 0.06	1.70 ± 0.49	712.33 ± 223.5	8.74 ± 0.11	12.60 ± 0.30	43.26 ± 0.64
EC 500	D 28	7.96 ± 2.02^*^	6.35 ± 0.31^*^	1134.00 ± 35.92	8.03 ± 1.43	14.75 ± 1.15	43.68 ± 2.63
	D 14	5.00 ± 1.22	3.00 ± 1.19	1280.33 ± 22.21	9.16 ± 0.14	14.26 ± 0.19	47.23 ± 0.64
EC 250	D 28	7.25 ± 0.66^*^	4.35 ± 1.12^*^	1139.00 ± 24.49	8.54 ± 0.40	13.05 ± 0.28	42.45 ± 0.44
	D 14	3.92 ± 1.71	3.15 ± 1.32	815.50 ± 412.64	9.16 ± 0.45	13.10 ± 0.48	45.65 ± 1.82
PS 500	D 28	5.81 ± 1.07^*^	4.85 ± 0.69^*^	1372.20 ± 163.95	9.26 ± 0.61	13.53 ± 1.02	48.78 ± 2.85
	D 14	2.00 ± 0.81	2.91 ± 1.12	1249.00 ± 230.98	8.31 ± 1.43	13.19 ± 1.77	49.57 ± 4.78
PS 250	D 28	5.66 ± 0.77^*^	2.99 ± 0.24^*^	1179.18 ± 88.67	9.55 ± 0.53	13.89 ± 0.24	47.40 ± 2.53
	D 14	3.30 ± 0.89	2.30 ± 0.88	1145.00 ± 212.35	7.43 ± 0.77	11.45 ± 0.88	40.90 ± 3.53

*Values are expressed in mean ± SD (n = 6/group)*.

* *Significant difference with respect to control at p < 0.05*.

#### Effect on blood biochemical parameters

Considering the KFTs and the LFTs, the administration of the mushroom extracts AL, EC, and CT caused significant changes in the parameters analyzed on both the 14th day as well as at the end of the study. The CRE, BUN, and TPR levels were found to be significantly higher than the control at both the doses of the extracts AL, EC, and CT (Table 2). Similar results were observed for ALT, AST, ALP, and TBIL in case of these mushrooms and the increase was noted to be dose dependent as shown in Table 3. However, with regard to the mushrooms CM, AE, PS, and AB, these values showed no significant increase in comparison to control.

**Table 2:** Effect of the mushroom extracts on kidney functioning on day 14 and 28.

		**CRE (mg/dL)**	**BUN (mg/dL)**	**TPR (mg/dL)**
CONTROL	D 28	0.32 ± 0.07	23.94 ± 3.34	5.21 ± 0.15
	D 14	0.34 ± 0.04	26.44 ± 4.59	4.78 ± 0.14
AB 500	D 28	0.28 ± 0.06	20.60 ± 1.45	4.87 ± 0.22
	D 14	0.25 ± 0.05	18.20 ± 1.10	4.94 ± 0.08
AB 250	D 28	0.24 ± 0.05	17.54 ± 1.48	4.83 ± 0.21
	D 14	0.22 ± 0.04	16.56 ± 2.15	4.36 ± 0.47
AE 500	D 28	0.44 ± 0.02	29.76 ± 1.23	5.69 ± 0.21
	D 14	0.33 ± 0.08	26.32 ± 2.69	5.41 ± 0.35
AE 250	D 28	0.40 ± 0.02	28.66 ± 1.49	5.81 ± 0.29
	D 14	0.28 ± 0.05	23.44 ± 1.06	4.98 ± 0.14
AL 500	D 28	0.58 ± 0.13^*^	41.23 ± 5.18^*^	6.79 ± 0.42^*^
	D 14	0.51 ± 0.02^*^	39.23 ± 4.40^*^	6.09 ± 0.19^*^
AL 250	D 28	0.54 ± 0.05^*^	40.09 ± 3.06^*^	6.71 ± 0.56^*^
	D 14	0.49 ± 0.01^*^	36.32 ± 2.66	5.97 ± 0.26^*^
CM 500	D 28	0.40 ± 0.05	29.73 ± 6.94	6.11 ± 0.62
	D 14	0.33 ± 0.01	25.34 ± 1.30	5.21 ± 0.15
CM 250	D 28	0.34 ± 0.02	28.51 ± 1.62	5.69 ± 0.21
	D 14	0.28 ± 0.05	22.32 ± 4.83	4.96 ± 0.06
CT 500	D 28	0.54 ± 0.05^*^	39.31 ± 5.98^*^	6.61 ± 0.46^*^
	D 14	0.45 ± 0.03^*^	35.34 ± 4.30	5.94 ± 0.28^*^
CT 250	D 28	0.34 ± 0.01	23.48 ± 3.97	5.64 ± 0.53
	D 14	0.28 ± 0.06	21.29 ± 2.16	4.93 ± 0.04
EC 500	D 28	0.57 ± 0.03^*^	38.95 ± 4.59^*^	6.56 ± 0.47^*^
	D 14	0.48 ± 0.01^*^	34.62 ± 3.89	5.84 ± 0.41^*^
EC 250	D 28	0.34 ± 0.02	28.48 ± 1.03	5.64 ± 0.53
	D 14	0.28 ± 0.05	20.94 ± 3.34	4.93 ± 0.09
PS 500	D 28	0.39 ± 0.02	29.44 ± 1.06	5.75 ± 0.42
	D 14	0.33 ± 0.02	24.57 ± 1.87	4.98 ± 0.26
PS 250	D 28	0.34 ± 0.01	28.48 ± 2.01	5.60 ± 0.45
	D 14	0.24 ± 0.07	20.60 ± 1.55	4.87 ± 0.27

*Values are expressed in mean ± SD (n = 6/group)*.

* *Significant difference with respect to control at p < 0.05*.

**Table 3:** Effect of the mushroom extracts on liver functioning on day 14 and 28.

		**AST (IU/L)**	**ALT (IU/L)**	**ALK (IU/L)**	**TBIL (mg/dL)**
CONTROL	D 28	56.58 ± 5.38	71.50 ± 8.63	119.52 ± 6.90	0.30 ± 0.01
	D 14	55.66 ± 4.92	78.62 ± 7.76	117.39 ± 12.66	0.33 ± 0.04
AB 500	D 28	63.32 ± 6.03	63.69 ± 3.91	130.28 ± 5.66	0.26 ± 0.02
	D 14	62.30 ± 1.81	60.78 ± 4.30	121.83 ± 7.33	0.29 ± 0.01
AB 250	D 28	60.95 ± 4.32	59.98 ± 7.86	120.86 ± 1.62	0.31 ± 0.04
	D 14	55.55 ± 2.04	54.97 ± 1.70	120.86 ± 12.31	0.20 ± 0.07
AE 500	D 28	74.48 ± 5.11	92.67 ± 5.11	155.45 ± 17.02	0.36 ± 0.05
	D 14	67.50 ± 5.03	85.80 ± 2.13	145.96 ± 16.51	0.33 ± 0.01
AE 250	D 28	76.4 ± 6.52	89.95 ± 4.55	143.24 ± 2.28	0.38 ± 0.05
	D 14	67.49 ± 3.91	65.53 ± 1.06	127.36 ± 3.50	0.26 ± 0.01
AL 500	D 28	118.11 ± 2.85^*^	162.07 ± 4.27^*^	266.28 ± 6.58^*^	0.86 ± 0.07^*^
	D 14	105.31 ± 6.85^*^	122.00 ± 8.90^*^	201.60 ± 7.96^*^	0.70 ± 0.04^*^
AL 250	D 28	100.62 ± 5.63^*^	130.96 ± 2.13^*^	195.80 ± 3.36^*^	0.72 ± 0.01^*^
	D 14	94.68 ± 6.94^*^	114.94 ± 9.61^*^	183.19 ± 8.75^*^	0.58 ± 0.02^*^
CM 500	D 28	78.64 ± 4.89	82.00 ± 10.26	161.60 ± 7.96	0.38 ± 0.05
	D 14	68.86 ± 10.37	79.86 ± 13.94	148.71 ± 19.83	0.36 ± 0.04
CM 250	D 28	71.42 ± 5.87	74.06 ± 10.90	144.23 ± 18.16	0.33 ± 0.06
	D 14	65.58 ± 11.07	73.29 ± 6.89	148.99 ± 21.29	0.28 ± 0.02
CT 500	D 28	109.68 ± 3.43^*^	146.45 ± 3.84^*^	254.66 ± 3.34^*^	0.76 ± 0.03^*^
	D 14	97.80 ± 1.79^*^	121.52 ± 2.13^*^	176.59 ± 5.72^*^	0.46 ± 0.03^*^
CT 250	D 28	61.95 ± 7.12	78.61 ± 7.42	140.99 ± 18.53	0.35 ± 0.06
	D 14	60.49 ± 3.91	62.68 ± 6.76	139.74 ± 11.72	0.27 ± 0.04
EC 500	D 28	113.11 ± 4.85^*^	165.24 ± 8.06^*^	252.73 ± 7.51^*^	0.83 ± 0.03^*^
	D 14	100.62 ± 5.63^*^	130.96 ± 2.13^*^	195.8 ± 3.36^*^	0.72 ± 0.01^*^
EC 250	D 28	79.80 ± 1.79	85.52 ± 5.56	176.59 ± 5.72	0.40 ± 0.03^*^
	D 14	71.25 ± 6.12	69.53 ± 1.06	137.81 ± 7.96	0.26 ± 0.04
PS 500	D 28	79.17 ± 5.32	78.27 ± 4.93	150.53 ± 6.62	0.38 ± 0.04
	D 14	68.66 ± 4.62	89.52 ± 6.04	153.74 ± 20.48	0.39 ± 0.07
PS 250	D 28	76.72 ± 10.88	84.20 ± 1.77	122.98 ± 7.54	0.48 ± 0.14
	D 14	68.70 ± 3.91	65.00 ± 10.48	116.94 ± 10.70	0.25 ± 0.04

*Values are expressed in mean ± SD (n = 6/group)*.

* *Significant difference with respect to control at p < 0.05*.

#### Histopathological analysis

**Figure 7:**
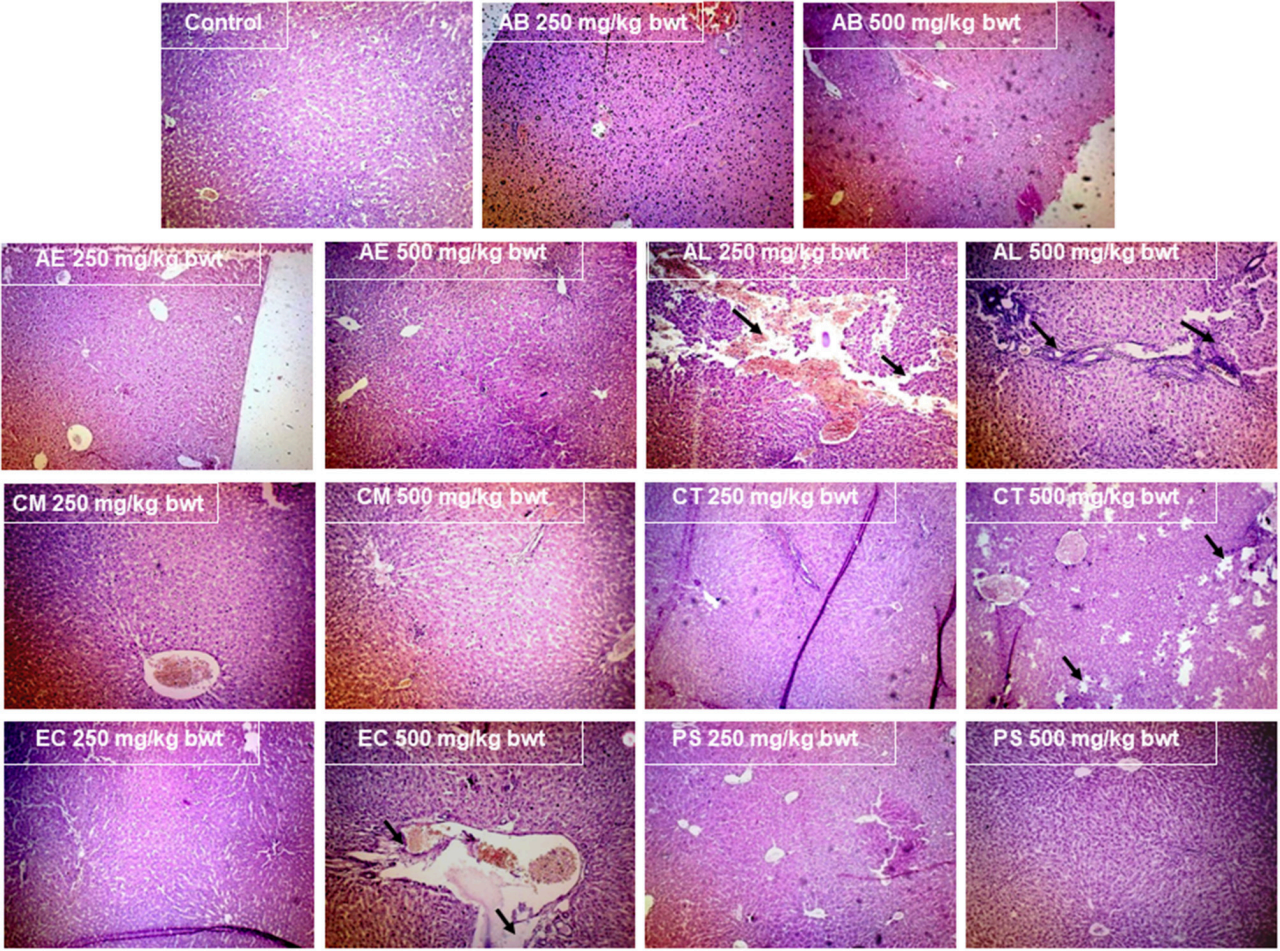
Effect of the mushroom extracts on the liver histology (Magnification 100x).

**Figure 8:**
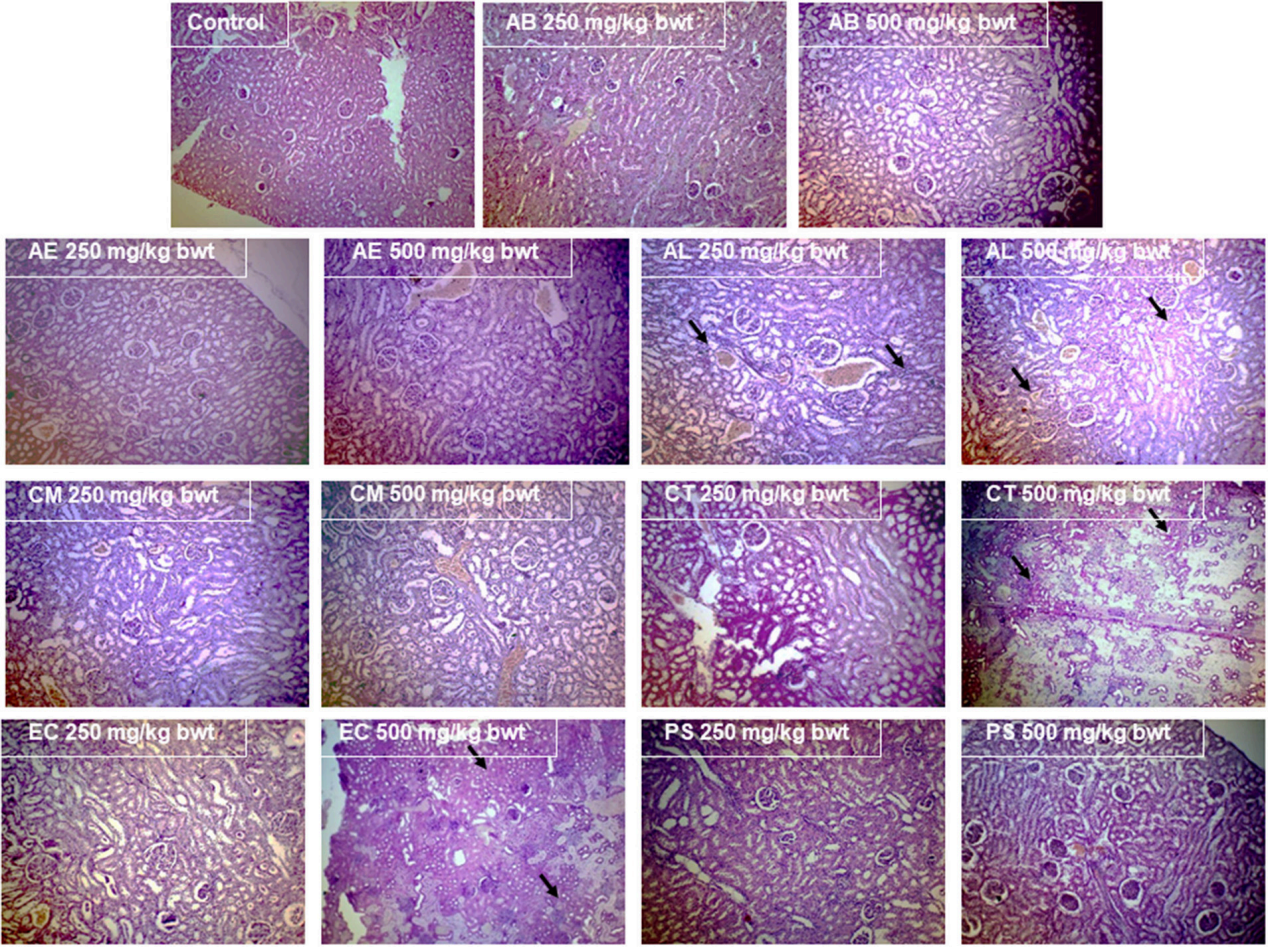
Effect of the mushroom extracts on the kidney histology (Magnification 100x).

**Figure 9:**
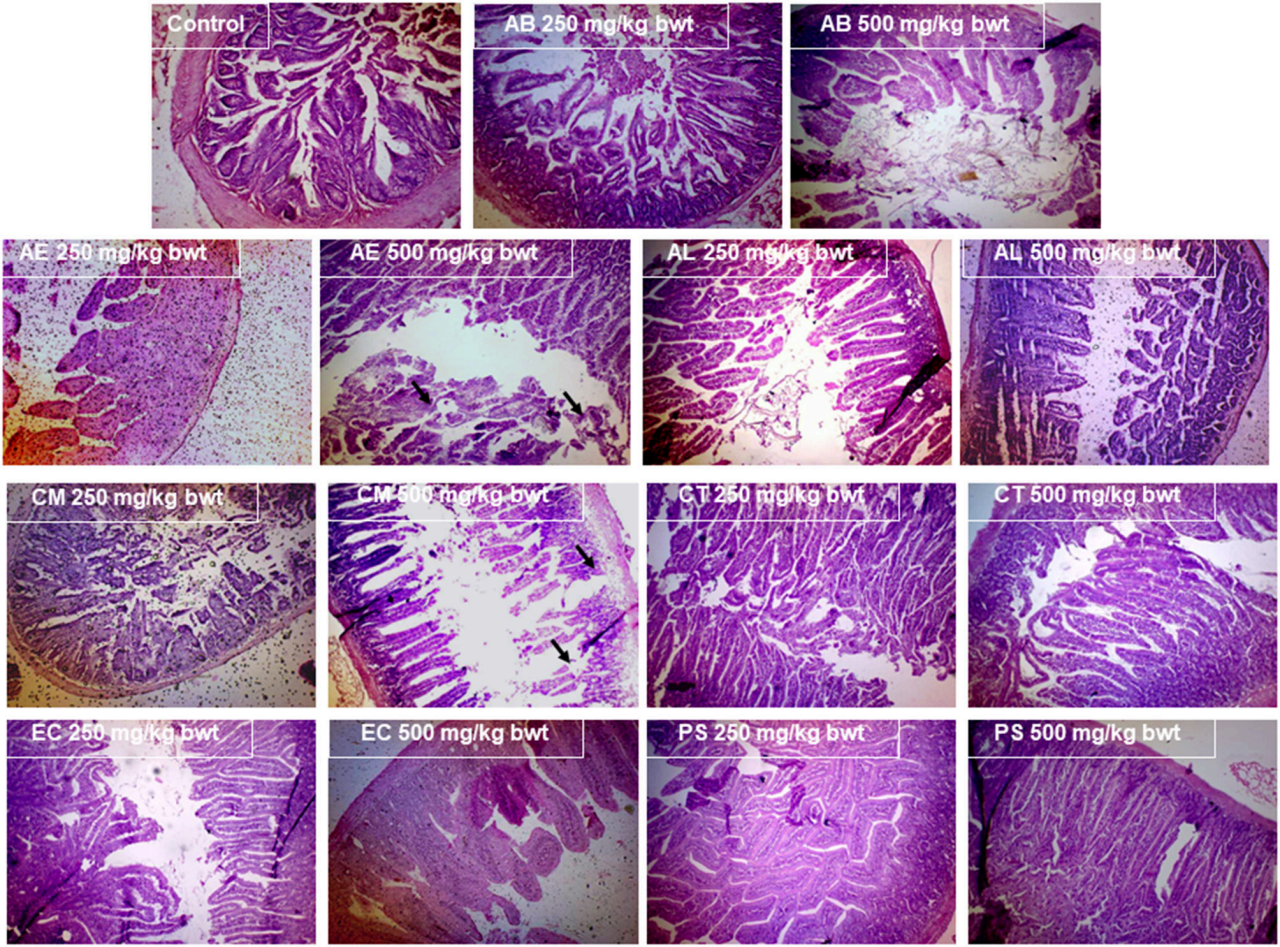
Effect of the mushroom extracts on the small intestine histology (Magnification 100x).

The examination of the liver (Figure 7) and kidney (Figure 8) tissues on sacrificing the animals at the end of the study period, revealed evident pathology on exposure to the mushrooms, AL, EC, and CT. In case of AL, a dose dependent damage was noted in the pathological signs exhibited in the liver, with the lower dose causing only mild feathery degeneration while there was additional damage of biliary stasis and the distortion of the tissue was to a greater extent at the higher dose. The exposure to 500 mg/kg bwt of the EC and CT extracts resulted in distortion of hepatic lobular architecture and degeneration of hepatocytic features respectively. Distorted glomerular and tubular morphology was a pathological observation of the kidney tissue, common to the mushrooms EC and CT at 500 mg/kg bwt. However, the administration of AL at the higher dose caused a chronic venous congestion and mild congestive features were observed at the lower dose as well. Although, no liver and kidney pathology was noticed in case of the other mushrooms, AE and CM extracts at the higher dose caused injury and sloughing of the mucosal layer of the small intestine (Figure 9). PS and AB extracts caused no pathological changes in the liver, kidney, or the small intestine. The mushroom extracts caused no significant pathological changes in the large intestine and the brain (Data not shown).

### GC-MS analysis

**Figure 10:**
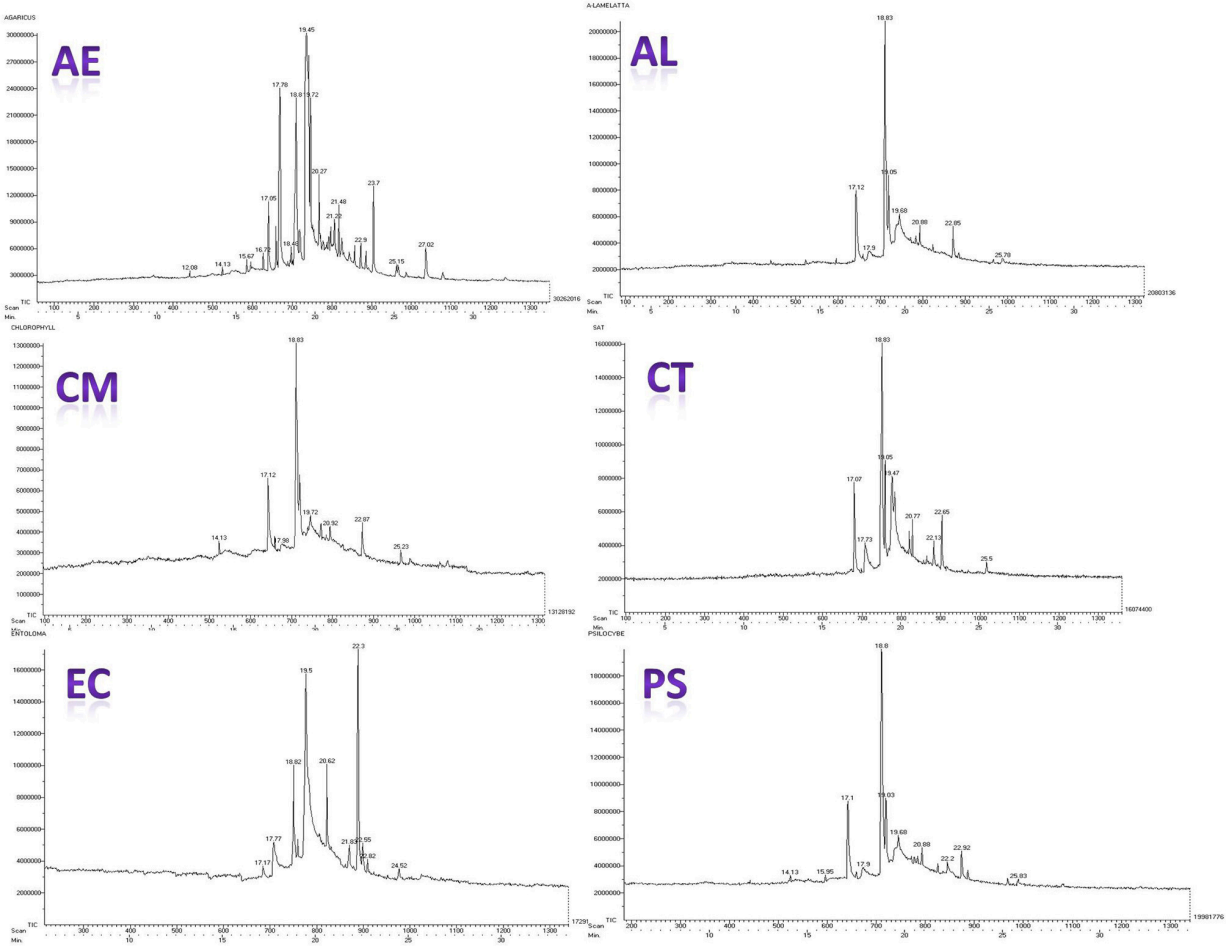
Gas chromatograms of the mushroom extracts.

Figure 10 presents the gas chromatograms of hydro-ethanolic extracts of the mushrooms under the study. The compounds present in these extracts were identified based on comparison of their retention times and molecular weight with reference compounds available in NIST library of GC-MS. Peak assignment was also confirmed by mass spectrometry. Further, molecular structure, and molecular formula were obtained based on the MS fragmentation pattern (Supplementary Tables 3–8). The compounds identified in the extracts can be divided broadly into the following chemical classes: (1) Esters of fatty acids (2) Alcohols (3) Ketones (4) Terpenes (5) Nitrogen containing compounds (6) Steroids (7) Phenols and flavonoids. These compounds constitute the volatile profile of the selected wild mushroom species.

## Discussion

### *In vitro* intestinal toxicity

For the preliminary *in vitro* study, the NCM460 colon epithelial cell line was selected to represent the intestinal barrier as these cells are the earliest to come in contact with mushrooms on their consumption. The toxic potential of the mushrooms was assessed considering both the effects on cell morphology and their IC_50_ values. Morphological changes and abnormalities such as cell shrinkage, vacuolation, and distortion in cell shape were evident in the cells exposed to the extracts as shown in Figure 2A, indicating a marked possibility of toxicity associated with the consumption of these mushrooms. Our finding of the intestinal toxicity associated with CM stands in agreement to the incidents of poisonings documented on consuming this mushroom which were generally accompanied by severe gastrointestinal symptoms (Young, 1989; Natarajan and Kaviyarasan, 1991; Benjamin, 1995). The observation that AE also showed a significantly low IC_50_ value (0.73 mg/mL) and caused adverse structural changes in intestinal cells is in line with the reports that members of its taxonomic section such as *A. xanthodermus* are associated with gastrointestinal disturbances (Sitotaw et al., 2016). The case of poisoning in Poland on consumption of *Entoloma sericeum* (Kapala et al., 2008) suggests that the *Entoloma* species might be responsible for the gastrointestinal syndrome. This possibility was also observed in our study wherein EC showed significant signs of intestinal toxicity (Figure 2A). In case of AL also, it was observed that there were distinct changes in the morphology of intestinal cells in comparison to the control cells. This may be of toxicological significance as generally the first phase of *Amanita* poisonings are characterized by acute episodes of gastrointestinal disturbances exhibited as profuse vomiting and diarrhea (Mitchel, 1980). The extracts of CT and PS showed minimal adverse effects on the intestinal cells, while the edible AB caused no significant changes and in effect was almost similar to that of the control cells, indicating its nontoxicity.

### *In vitro* hepatotoxicity

Liver being both the target for as well as an organ of defense against the exogenous compounds, an *in vitro* toxicity evaluation using the liver cell line was considered in the study. Cell viability was monitored to assess the overall effect on the cells. As evident from the Figure 3, the mushroom extracts exhibited potential hepatotoxicity *in vitro*. Biologically relevant endpoints besides cell viability such as membrane integrity, mitochondrial stress, and oxidative status were also measured in the hepatocytes exposed to the extracts. Among the mushrooms studied, AL was found to be the most toxic to the hepatocytes. Similar results of *in vitro* hepatotoxicity have been demonstrated on exposure to toxic *Amanita* species such as *A. abrupta, A.virosa*, and *A.volvata* (Kawaji et al., 1990). Also, AL which showed relatively the least IC_50_ among the mushrooms caused the maximum damage to the membrane which was reflected in the high levels of leaked LDH (Figures 3B, 4). This observation is in consonance with the principle that leakage in cytosolic LDH increases with increase in the number of dead cells (Legrand et al., 1992). Furthermore, the study clearly demonstrated a significant increase in the trigger of ROS and NO generation in the presence of the extracts in comparison to the control. It has also been shown that an accumulation of these reactive species generally causes lipid peroxidation leading to an increase in the permeability of mitochondrial membrane and a consequent collapse in the mitochondrial membrane potential (Ott et al., 2007). Our study has shown to be consistent with this understanding as the results point out that the mushrooms causing the highest generation of oxidative stress resulted in relatively lower mitochondrial membrane potentials (Figures 6B,D).

The hepatotoxicity observed due to CT and EC is a new finding and adds to their scanty literature. Although the hepatotoxicity due to these mushrooms was significant, it was relatively milder in comparison to other mushrooms. Comparing these results to their effects on the NCM460 colon epithelial cells, it can possibly be inferred that these two mushrooms affect the intestine to a greater extent than the liver. In the present study, PS was observed to be least toxic to both the intestinal cells as well as the hepatocytes. This result is strongly evidenced in the literature wherein members of the *Psilocybe* genus are mostly associated with complications of the hallucinogenic nature (Peden et al., 1981) and thus could have probably been associated with minimum cytotoxic effects on the intestine and liver.

### *In vivo* toxicity

In order to examine the *in vivo* systemic toxicity and identify the organ(s) targeted by mushroom extracts, study was carried out using the mice model and female ones were preferred as they are generally slightly more sensitive to toxic effects (Lipnick et al., 1995). As the most significant human exposure to mushrooms occurs through ingestion, the oral route was chosen for the *in vivo* study which aimed to assess the potential effect of consuming these mushrooms. Since the yield on extraction was about 3.5% of the fresh weight of the mushrooms, the dosage of 500 mg mushroom extract/kg body weight (bwt) for the mice when extrapolated to humans is approximately equivalent to one serving of mushroom per meal (70 g; Conversion factor of 1/12; Reagan-Shaw et al., 2008). A preliminary study was conducted wherein single doses of the extracts were administered at 500 mg/kg bwt to different groups of female mice (*n* = 3) in order to verify the acute toxicity. However, no mortality or adverse changes were observed at the end of the study period of 14 days (Data not shown). In the cases of mushroom poisoning that have been recorded, the victims have shown differential responses with respect to the time for the onset of symptoms as well as the dose required to cause the effect. In many cases, a repeated exposure to the mushrooms through consecutive meals had resulted in the manifestation of the symptoms (Nieminen et al., 2008). Therefore, the OECD guideline TG 407 (OECD, 2008) for 28 day sub-acute toxicity was followed at dosages of the mushroom extracts which had relevance to the daily intake of mushrooms by humans. There were no marked behavioral changes observed except for the group of animals exposed to PS. These behavioral responses have also been exhibited by those administered with *P. cubensis* (Kirsten and Bernardi, 2010), suggesting that these symptoms may be characteristic of hallucinogenic species of the *Psilocybe* genus. Taking into account the hematological parameters, the increased levels of LYM and WBC may serve as putative markers of an inflammatory response and also helps in predicting toxicity or pathological status. In addition to the hematological parameters, the clinical biochemistry data also plays an important role in determining the toxicity induced by various drugs and chemical substances. The biochemical parameters such as ALP, ALT, AST, and total bilirubin are indicators of hepatic functions; while total protein, creatinine, and urea levels are good measures of the renal functions (Kong et al., 2016). ALT and AST present in the cytoplasm of hepatocytes show a marked elevation in their activities in the bloodstream on account of any liver injury and are sensitive markers of liver tissue damage (Ramaiah, 2007). ALP which is also particularly concentrated in the liver shows an increase on account of distortion in the hepatic architecture. The results of this study showed that AL 250, AL 500, CT 500, EC 500 caused significant increase in the levels of the parameters that reflect the kidney and liver functioning assessed on day 14. Also as mentioned previously, the administration of the extracts except for PS caused a body weight reduction in the animals on the day 14. These observations indicate that there are obvious adverse effects on short term consumption of these mushrooms at relevant human dosages.

As mushroom poisonings are generally associated with gastrointestinal, renal, hepatic, or neurological effects (de Oliveira, 2009), the small and large intestines, kidney, liver, and brain were examined for any pathological signs. Interestingly, the inferences of the clinical biochemistry data besides having toxicological relevance substantiate the pathological findings indicating that liver and kidneys were the target organs of the mushrooms AL, EC, and CT. The significant increase in the serum bilirubin which serves as a prognostic factor in conditions leading to hepatocellular necrosis (McDonald et al., 1993), was particularly of great significance for AL which showed marked biliary stasis (Figure 7). This finding also stands in good agreement with the well-established fact that liver injury has been associated with many cases of *Amanita* poisonings (Bartoloni St Omer et al., 1985). The pathological effects of these mushrooms were also observed in the kidneys, characterizing their toxicity as possible hepatorenal syndrome. There were also congestive features and disruption in the glomerular and tubular ultrastructure (Figure 8) as was reflected in the elevated levels of serum creatinine, urea, and protein. There have been poisoning reports of renal insufficiency and hepatic damage associated with a few *Amanita* species such as *A. regalis* (Elonen et al., 1979) which support our findings with respect to AL.

The sloughing of the luminal mucosa with mild inflammation of the intestine in case of the mushrooms CM and AE was in consonance with our *in vitro* toxicity results. This finding may also prove a useful evidence in explaining the case of severe intestinal abnormalities reported on consumption of CM in Kerala (Bijeesh et al., 2017). In the poisonings reported in humans on account of *C. molybdites* (CM) and other species of the section *Xanthodermatei* (to which AE belongs), early symptoms such as vomiting and nausea were recorded within few hours of their consumption. However, such immediate effects cannot be observed in mice due to their inability to exhibit emesis. But their intestinal toxicity was evident in the pathological observations. Therefore, this study suggests that these mushroom species are best avoided. There were no marked pathological changes noted in the organs of the animals exposed to PS and this again reinstates the *in vitro* results which indicate its relatively low toxicity. The toxic potential of these mushrooms indicated by the findings of this study, though predictive in nature warrants caution about their edibility and also suggests a more detailed investigation in ascertaining their toxic properties.

## Conclusion

Through this study, we may speculate the toxic properties of these wild mushrooms and hazard an opinion as to their probable effect on consumption by man. For the clinical treatment of a victim of mushroom poisoning, it is essential to identify the mushroom species. But in most cases, the information of the causative mushroom is unavailable due to reasons of having been consumed or disposed at the time of treatment, and hence the species are estimated by their clinical symptoms. Therefore, the biochemical information from the blood and serum gathered from this study may also serve diagnostic purposes in cases of accidental poisonings by these mushrooms. The mushroom species under the study although have been harvested locally, their occurrence has also been reported in other parts of the world (Supplementary Table 9). Therefore, this study which has attempted to explore the edibility of the representative mushrooms from the wild warrants caution about consuming these species elsewhere in the world too. Moreover, global researchers working on mycetism will also be befitted with the current study. Hence, the present work could have broader implications for global mycetism. Also, identifying the toxic compound(s) responsible for and the mechanism of the observed toxicity of these wild mushrooms deserves further exploration and a detailed evaluation.

## Author contributions

SS, SN, CP, CS, MD, KA: The conception or design of the work; or the acquisition, analysis, or interpretation of data for the work; Drafting the work or revising it critically for important intellectual content; Final approval of the version to be published; Agreement to be accountable for all aspects of the work in ensuring that questions related to the accuracy or integrity of any part of the work are appropriately investigated and resolved.

### Conflict of interest statement

The authors declare that the research was conducted in the absence of any commercial or financial relationships that could be construed as a potential conflict of interest.
