# Supplementary material for: Toxicity Assessment of Wild Mushrooms from the Western Ghats, India: An in Vitro and Sub-Acute in Vivo Study
Source: Front Pharmacol. 2018 Feb 13;9:90. doi: 10.3389/fphar.2018.00090 (PMC5816808; doi:10.3389/fphar.2018.00090)
Supplement: Supplementary file 1 [file Table1.docx]

| **Sl.No.** | **MUSHROOM SPECIES** | **CLASSIFICATION** | **EXPCTED TOXIN** | **EXPECTED TOXICITY** |
| --- | --- | --- | --- | --- |
| **1.** | *Amanita angustilamellata (AL)* | Group 1 | Amatoxins | Hepatotoxicity |
| **2.** | *Psilocybe subcubensis (PS)* | Group 6 | Psilocybin | Hallucinogenic effects |
| **3.** | *Agaricus endoxanthus (AE)* | Group 7 | Gastrointestinal irritants | Gastrointestinal  disturbances |
| **4.** | *Chlorophyllum molybdites (CM)* | Group 7 | Gastrointestinal irritants | Gastrointestinal  disturbances |
| **5.** | *Clarkeinda trachodes (CT)* | Group 7 | Unknown | Unknown |
| **6.** | *Entoloma crassum (EC)* | Group 7 | Unknown | Unknown |

**Table 1- Classification of the mushroom species under the study according to**

**POISINDEX Information System**
