# Supplementary material for: Toxicity Assessment of Wild Mushrooms from the Western Ghats, India: An in Vitro and Sub-Acute in Vivo Study
Source: Front Pharmacol. 2018 Feb 13;9:90. doi: 10.3389/fphar.2018.00090 (PMC5816808; doi:10.3389/fphar.2018.00090)
Supplement: Supplementary file 2 [file Table2.docx]

| **Sl.No.** | **INGREDIENTS** | **COMPOSITION (%)** |
| --- | --- | --- |
| 1 | Wheat flour | 15 |
| 2 | Roasted Bengalgram dhal | 58 |
| 3 | Groundnut flour | 10 |
| 4 | Skim milk powder | 5 |
| 5 | Casein | 4 |
| 6 | Refined oil | 4 |
| 7 | Salt mixture | 4 |
| 8 | Vitamin mixture | 0.2 |

**Table 2- Composition of stock diet for mice**
