# Supplementary material for: Toxicity Assessment of Wild Mushrooms from the Western Ghats, India: An in Vitro and Sub-Acute in Vivo Study
Source: Front Pharmacol. 2018 Feb 13;9:90. doi: 10.3389/fphar.2018.00090 (PMC5816808; doi:10.3389/fphar.2018.00090)
Supplement: Supplementary file 3 [file Table3.DOCX]

| **SL.NO** | **RT** | **NAME** | **IUPAC NAME** | **MOL.WT**  **(g/mol)** | **MOL. FORMULA** | **STRUCTURE** | **REFERENCE NUMBER** |
| --- | --- | --- | --- | --- | --- | --- | --- |
| 1. | 12.08 | 2-Buten-1-ol,3 methyl | 3-methyl-2-butenol | 86.132 | C_5_H_10_O | 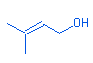 | NIST CAS # 556-82-1 #ions=51 |
| 2. | 14.13 | 3-Buten-2-one, 4-(2,6,6-trimethyl-2-cyclohexen-1-yl)-,(E)- | 4-(2,6,6-Trimethyl-2-cyclohexenyl)-3-buten-2-one | 192.297 | C_13_H_20_O | 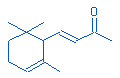 | NIST CAS # 127-41-3 #ions=136 |
| 3. | 15.67 | Tetradecanoic acid, ethyl ester | **Ethyl myristate** | 256.424 | C_16_H_32_0_2_ | 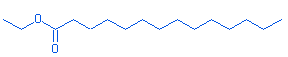 | NIST MS 1 OF 100  (124-06-1) #ions=120 |
| 4. | 16.72 | Pentadecanoic acid, ethyl ester | Ethyl pentadecanoate | 270.457 | C_17_H_34_0_2_ | 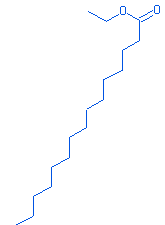 | NIST MS 1 OF 100  (41114-00-) #ions=131 |
| 5. | 17.05 | Pentadecanoic acid, 14-methyl-methyl ester | Methyl 14-methylpentadecanoate | 270.457 | [C_17_H_34_O_2_](https://pubchem.ncbi.nlm.nih.gov/search/#collection=compounds&query_type=mf&query=C17H34O2&sort=mw&sort_dir=asc) | 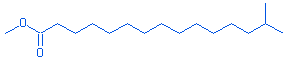 | NIST MS 2 OF 100  (5129-60-2 #ions=104 |
| 6. | 17.78 | Hexadecanoic acid, ethyl ester | **Ethyl palmitate** | 284.477 | C_18_H_36_O_2_ | 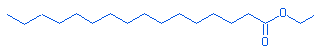 | NIST MS 1 OF 100  (628-97-7) #ions=170 |
| 7. | 18.48 | Ethanol,2-(9,12-Octadecadienyloxy)-, (Z,Z)- | **2-[(9Z,12Z)-9,12-Octadecadien-1-yloxy]ethanol** | 310.514 | C_20_H_38_O_2_ | 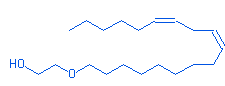 | NIST MS 6 OF 100  (17367-08- #ions=263 |
| 8. | 18.8 | 10-Octadecenoic acid, methyl ester | **Methyl (10E)-10-octadecenoate** | 296.488 | C_19_H_36_O_2_ | 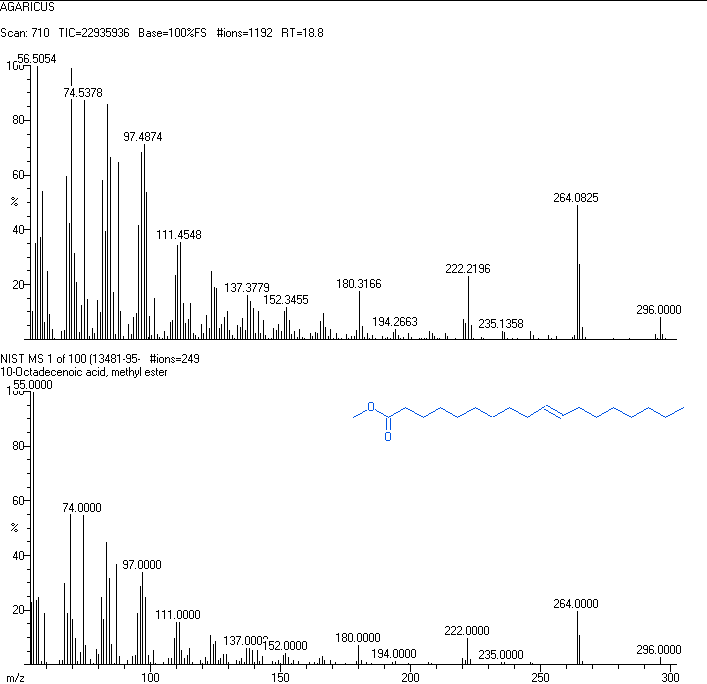 | NIST MS 1 OF 100  (13481-95- #ions=249 |
| 9. | 19.45 | 9,12-Octadecadienoic acid,ethyl ester | **Ethyl (9Z,12Z)-9,12-octadecadienoate** | 308.506 | [C_20_H_36_O_2_](https://pubchem.ncbi.nlm.nih.gov/search/#collection=compounds&query_type=mf&query=C18H32O2&sort=mw&sort_dir=asc) | 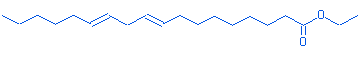 | NIST MS 1 OF 100  (7619-08-1 #ions=134 |
| 10. | 19.72 | Heptadecanoic acid, 15-methyl, ethyl ester | **Ethyl 15-methylheptadecanoate** | 312.541 | C_20_H_40_O_2_ | 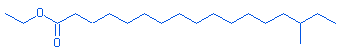 | NIST MS 1 OF 100  (57274-46- #ions=148 |
| 11. | 20.27 | Isopropyl linoleate | **Isopropyl (9Z,12Z)-9,12-octadecadienoate** | 322.533 | [C_21_H_38_O_2_](https://pubchem.ncbi.nlm.nih.gov/search/#collection=compounds&query_type=mf&query=C21H38O2&sort=mw&sort_dir=asc) | 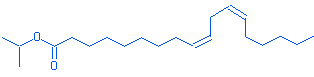 | NIST MS 8 OF 100  (22882-95- #ions=150 |
| 12. | 21.22 | Cyclopropaneoctanoic acid, 2((2-((2-ethylcyclopropyl)methyl)cyclopropyl) methyl methyl ester) | - | 334.53 | C_22_H_38_O_2_ | 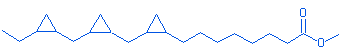 | NIST MS 3 OF 100  (10152-71- #ions=157 |
| 13. | 21.48 | Octadecyl methacrylate | octadecyl 2-methylprop-2-enoate | 338.576 | [C_22_H_42_O_2_](https://pubchem.ncbi.nlm.nih.gov/search/#collection=compounds&query_type=mf&query=C22H42O2&sort=mw&sort_dir=asc) | 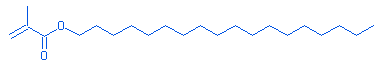 | NIST MS 100 OF 100  (112-08- #ions=193 |
| 14. | 22.9 | Docosanoic acid, methyl ester | Methyl docosanoate | 354.619 | [C_23_H_46_O_2_](https://pubchem.ncbi.nlm.nih.gov/search/#collection=compounds&query_type=mf&query=C23H46O2&sort=mw&sort_dir=asc) | 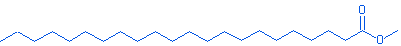 | NIST MS 1 OF 100  (929-77-1) #ions=191 |
| 15. | 23.7 | Docosanoic acid, ethyl ester | Ethyl docosanoate | 368.646 | [C_24_H_48_O_2_](https://pubchem.ncbi.nlm.nih.gov/search/#collection=compounds&query_type=mf&query=C24H48O2&sort=mw&sort_dir=asc) | 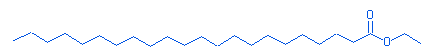 | NIST MS 1 OF 100  (5908-87-2) #ions=161 |
| 16. | 25.15 | 9-octadecenoic acid (Z), 2-butoxyethyl ester | **2-Butoxyethyl (9Z)-9-octadecenoate** | 382.620 | C_24_H_46_O_3_ | 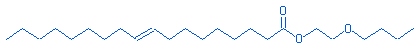 | NIST MS 31 OF 100  (109-39-7 #ions=230 |
| 17. | 27.02 | Ethyl tetra cosanoate | Ethyl tetracosanoate | 396.7 | [C_26_H_52_O_2_](https://pubchem.ncbi.nlm.nih.gov/search/#collection=compounds&query_type=mf&query=C26H52O2&sort=mw&sort_dir=asc) | 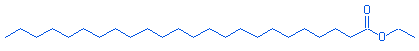 | NIST MS 2 OF 100  (24634-95- #ions=187 |

**Table 3-Compounds present in *Agaricus endoxanthus* (AE) extract analysed using GC-MS**
