# Supplementary material for: Toxicity Assessment of Wild Mushrooms from the Western Ghats, India: An in Vitro and Sub-Acute in Vivo Study
Source: Front Pharmacol. 2018 Feb 13;9:90. doi: 10.3389/fphar.2018.00090 (PMC5816808; doi:10.3389/fphar.2018.00090)
Supplement: Supplementary file 4 [file Table4.DOCX]

| **SL.NO** | **RT** | **NAME** | **IUPAC NAME** | **MOL.WT**  **(g/mol)** | **MOL. FORMULA** | **STRUCTURE** | **REFERENCE**  **NUMBER** |
| --- | --- | --- | --- | --- | --- | --- | --- |
| 1. | 14.13 | Triethanolamine | **2,2',2''-Nitrilotriethanol** | 149.188 | C_6_H_15_NO_3_ | 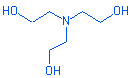 | NIST CAS # 102-71-6 #ions=55 |
| 2. | 17.12 | Hexadecanoic acid, methyl ester | **Methyl palmitate** | 270.451 | [C_17_H_34_O_2_](https://pubchem.ncbi.nlm.nih.gov/search/#collection=compounds&query_type=mf&query=C16H32O2&sort=mw&sort_dir=asc) | 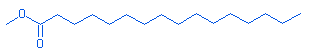 | NIST MS 7 OF 100  (112-38-0) #ions=152 |
| 3. | 17.98 | 4H-1-Benzopyran-4-one, 5,7-dihydroxy-3-phenyl | **5,7-Dihydroxy-2-phenyl-4H-chromen-4-one** | 254.238 | C_15_H_10_O_4_ |  | NIST CAS # 4044-00-2 #ions=35 |
| 4. | 18.83 | Phytol | (2E,7R,11R)-3,7,11,15-tetramethylhexadec-2-en-1-ol | 296.539 | [C_20_H_40_O](https://pubchem.ncbi.nlm.nih.gov/search/#collection=compounds&query_type=mf&query=C20H40O&sort=mw&sort_dir=asc) | 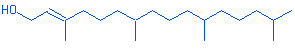 | NIST CAS # 150-86-7 #ions=166 |
| 5. | 19.72 | Oleic acid | **(9Z)-9-Octadecenoic acid** | 282.468 | C_18_H_34_O_2_ | **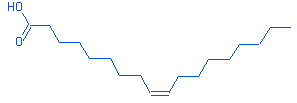** | NIST MS 7 OF 100  (112-80-1) #ions=247 |
| 6. | 20.92 | Corynan-17ol,18,19-didehydro-10-methoxy- | 2-(3-ethenyl-9-methoxy-1,2,3,4,6,7,12,12b-octahydroindolo[2,3-a]quinolizin-2-yl)ethyl acetate | 368.477 | [C_22_H_28_N_2_O_2_](https://pubchem.ncbi.nlm.nih.gov/search/#collection=compounds&query_type=mf&query=C22H28N2O3&sort=mw&sort_dir=asc) |  | NIST MS 9 OF 100  (56053-12- #ions=238 |
| 7. | 22.87 | Heneicosanoic acid, 20-oxo, methyl ester | Methyl 20-oxohenicosanoate | 354.575 | [C_22_H_42_O_3_](https://pubchem.ncbi.nlm.nih.gov/search/#collection=compounds&query_type=mf&query=C22H42O3&sort=mw&sort_dir=asc) | 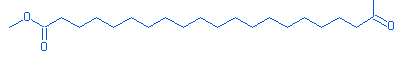 | NIST MS 31 OF 100  (56247-70 #ions=126 |
| 8. | 25.78 | Eicosanebioic acid, dimethyl ester | - | 370.00 | [C_22_H_42_O](https://pubchem.ncbi.nlm.nih.gov/search/#collection=compounds&query_type=mf&query=C22H42O3&sort=mw&sort_dir=asc)_4_ | 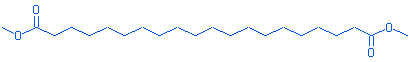 | NIST MS 5 OF 100  (42235-38- #ions=308 |

**Table 4-Compounds present in *Chlorophyllum molybdites* (CM) extract analysed using GC-MS**
