# Supplementary material for: Toxicity Assessment of Wild Mushrooms from the Western Ghats, India: An in Vitro and Sub-Acute in Vivo Study
Source: Front Pharmacol. 2018 Feb 13;9:90. doi: 10.3389/fphar.2018.00090 (PMC5816808; doi:10.3389/fphar.2018.00090)
Supplement: Supplementary file 5 [file Table5.DOCX]

| **SL.NO** | **RT** | **NAME** | **IUPAC NAME** | **MOL.WT**  **(g/mol)** | **MOL. FORMULA** | **STRUCTURE** | **Reference number** |
| --- | --- | --- | --- | --- | --- | --- | --- |
| 1. | 17.07 | 4’,5,7-Trihydroxy isoflavone | **5,7-Dihydroxy-3-(4-hydroxyphenyl)-4H-chromen-4-one** | 270.237 | C_15_H_10_O_5_ |  | NIST CAS # 446-72-0  #ions=145 |
| 2. | 17.73 | Estra-1,3,5 (10)-trien-17a’-ol | - | 256.00 | C_18_H_24_O | 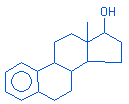 | NIST MS 5 OF 100  (2529-64-8) #ions=168 |
| 3. | 18.83 | 9-octadecenoic acid (Z)-, methyl ester | **Methyl (9Z)-9-octadecenoate** | 296.488 | C_19_H_36_O_2_ | 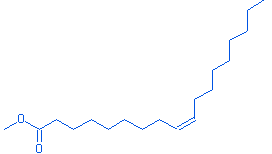 | NIST MS 1 OF 100  (112-62-9) #ions=225 |
| 4. | 19.05 | Octadecanoic acid, methyl ester | Methyl octadecanoate/  Methyl stearate | 298.511 | C_19_H_38_O_2_ | 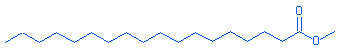 | NIST MS 1 OF 100  (112-61-8) #ions=107 |
| 5. | 19.47 | 6-octadecenoic acid, (Z)- | - | 282.00 | C_18_H_34_O_2_ | 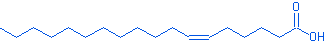 | NIST MS 7 OF 100  (593-39-5) #ions=230 |
| 6. | 20.77 | Eicosanoic acid, methyl ester | Methyl icosanoate | 326.565 | C_21_H_42_O_2_ | 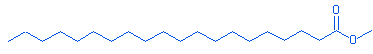 | NIST MS 19 OF 100  (1120-28- #ions=151 |
| 7. | 22.13 | Ethanol,2-(9-octadecenyloxy)-,(Z)- | **2-[(9Z)-9-Octadecen-1-yloxy]ethanol** | 312.530 | C_20_H_40_O_2_ | 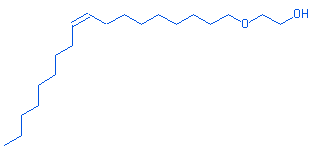 | NIST MS 5 OF 100  (5353-253 #ions=261 |
| 8. | 22.65 | Estra-1,3,5 (10),6-tetraene-3,17-diol,diacetate,(17a’) | - | 354.00 | C_21_H_38_O_4_ |  | NIST MS 13 OF 464  (1971-65- #ions=150 |
| 9. | 25.5 | 4 Hydroxy-3,5-dimethyl-6-(4-(2-methyl-3-(p-nitrophenyl)-2-propenylidene)tetrahydro-2-furyl) 2-pyranone | - | 383.00 | C_20_H_33_O_6_N |  | NIST MS 15 OF 100 (96265-34 #ions=325 |

**Table 5-Compounds present in *Clarkeinda trachodes* (CT) extract analysed using GC-MS**
