# Supplementary material for: Toxicity Assessment of Wild Mushrooms from the Western Ghats, India: An in Vitro and Sub-Acute in Vivo Study
Source: Front Pharmacol. 2018 Feb 13;9:90. doi: 10.3389/fphar.2018.00090 (PMC5816808; doi:10.3389/fphar.2018.00090)
Supplement: Supplementary file 6 [file Table6.DOCX]

| **SL.NO** | **RT** | **NAME** | **IUPAC NAME** | **MOL.WT**  **(g/mol)** | **MOL. FORMULA** | **STRUCTURE** | **Reference number** |
| --- | --- | --- | --- | --- | --- | --- | --- |
| 1. | 17.12 | Hexadecanoic acid, methyl ester | **Methyl palmitate** | 270.451 | C_17_H_34_O_2_ | **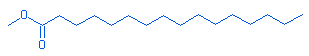** | NIST MS 1 OF 100  (112-39-0) #ions=152 |
| 2. | 17.9 | Estra-1,3,5(10)-trien-17a’-ol | 17a’-Estradiol, 3-deoxy- | 256.382 | C_18_H_24_O | 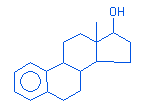 | NIST MS 4 OF 100  (2529-64-8) #ions=168 |
| 3. | 18.83 | 8-octadecenoic acid, methyl ester | **Methyl (8E)-8-octadecenoate** | 296.495 | [C_19_H_36_O_2_](https://pubchem.ncbi.nlm.nih.gov/search/#collection=compounds&query_type=mf&query=C19H36O2&sort=mw&sort_dir=asc) | 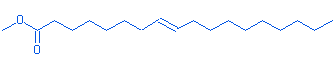 | NIST MS 1 OF 100  (2345-29-1 #ions=239 |
| 4. | 19.05 | Octadecanoic acid, methyl ester | Methyl octadecanoate | 298.511 | [C_19_H_38_O_2_](https://pubchem.ncbi.nlm.nih.gov/search/#collection=compounds&query_type=mf&query=C19H38O2&sort=mw&sort_dir=asc) | 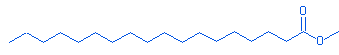 | NIST MS 1 OF 100  (112-61-8) #ions=107 |
| 5. | 19.68 | Oleic acid | **(9Z)-9-Octadecenoic acid** | 282.468 | C_18_H_34_O_2_ | 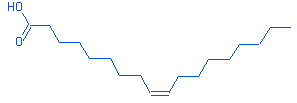 | NIST MS 1 OF 100 (112-80-1) #ions=247 |
| 6. | 20.88 | Eicosanoic acid, methyl ester | Methyl icosanoate | 326.565 | C_21_H_42_O_2_ | 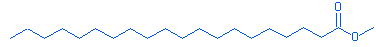 | NIST MS 10 OF 100  (1120-28- #ions=151 |
| 7. | 22.85 | Docosanoic acid, methyl ester | Methyl docosanoate | 354.619 | C_23_H_46_O_2_ | 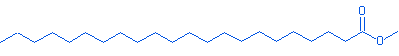 | NIST MS 3 OF 100  (929-77-1) #ions=191 |
| 8. | 25.78 | Pagicerine | **Methyl (19Z)-3-oxo-17,22-epoxyvobasan-16-carboxylate** | 380.437 | [C_22_H_24_N_2_O_4_](https://pubchem.ncbi.nlm.nih.gov/search/#collection=compounds&query_type=mf&query=C22H24N2O4&sort=mw&sort_dir=asc) | **** | NIST MS 6 OF 100  (99831-97- #ions=294 |

**Table 6-Compounds present in *Amanita angustilamellata* (AL) extract analysed using GC-MS**
