# Supplementary material for: Toxicity Assessment of Wild Mushrooms from the Western Ghats, India: An in Vitro and Sub-Acute in Vivo Study
Source: Front Pharmacol. 2018 Feb 13;9:90. doi: 10.3389/fphar.2018.00090 (PMC5816808; doi:10.3389/fphar.2018.00090)
Supplement: Supplementary file 7 [file Table7.DOCX]

| **SL.NO** | **RT** | **NAME** | **IUPAC NAME** | **MOL.WT**  **(g/mol)** | **MOL. FORMULA** | **STRUCTURE** | **REFERENCE NUMBER** |
| --- | --- | --- | --- | --- | --- | --- | --- |
| 1. | 17.17 | α-Pinene | **2,6,6-Trimethylbicyclo[3,1,1]hept-2-ene** | 136.238 | C_10_H_16_ | 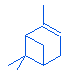 | NIST CAS # 80-56-8 #ions=71 |
| 2. | 17.77 | Coumarine, 7-formyl-4-methyl | **4-Methyl-2-oxo-2H-chromene-7-carbaldehyde** | 188.179 | C_11_H_8_O_3_ |  | NIST CAS # 53183-53-2 #ions=97 |
| 3. | 18.82 | Flavone | **2-Phenyl-4H-chromen-4-one** | 222.243 | C_15_H_10_O_2_ |  | NIST CAS # 525-82-6 #ions=78 |
| 4. | 19.5 | Oleic acid | **(9Z)-9-Octadecenoic acid** | 282.468 | C_18_H_34_O_2_ | 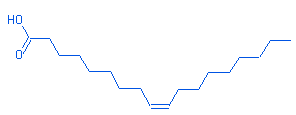 | NIST MS 1 OF 100  (112-80-1) #ions=247 |
| 5. | 20.62 | Hexadecanoic acid,1,1-dimethyl ethyl ester | **Ethyl 1,1-dimethylhexadecanoate** | 312.530 | C_20_H_40_O_2_ | 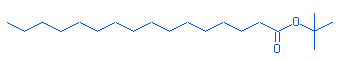 | NIST MS 33 OF 100 (31158-91 #ions=140 |
| 6. | 21.83 | 2,3-Dihydroxypropyl elaidate | **2,3-Dihydroxypropyl (9E)-9-octadecenoate** | 356.540 | C _21_H_40_O_4_ |  | NIST MS 3 OF 100  (2716-53-2 #ions=280 |
| 7. | 22.3 | (Z)-9-Octadecenoic acid butyl ester | **Butyl (9Z)-9-octadecenoate** | 338.568 | C_22_H_42_O_2_ |  | NIST MS 18 OF 100  (142-77-8) #ions=263 |
| 8. | 22.55 | Flavone 2',3,5,7-tetramethoxy- | 3,5,7-trimethoxy-2-(2-methoxyphenyl)chromen-4-one | 342.347 | C_19_H_18_O_6_ |  | NIST CAS # 14585-15-0 #ions=45 |
| 9. | 22.82 | Pentacosanoic acid, methyl ester | Methyl pentacosanoate | 396.7 | C_26_H_52_O_2_ | 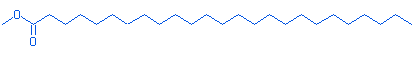 | NIST MS 10 OF 100 (55373-89 #ions=303 |
| 10. | 24.52 | Heptadecanoic acid, docosyl ester | Docosylheptadecanoate | 579.051 | [C_39_H_78_O_2_](https://pubchem.ncbi.nlm.nih.gov/search/#collection=compounds&query_type=mf&query=C39H78O2&sort=mw&sort_dir=asc) | 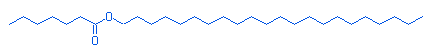 | NIST MS 1 OF 100  (55320-07- #ions=282 |

**Table 7-Compounds present in *Entoloma crassum* (EC) extract analysed using GC-MS**
