# Supplementary material for: Toxicity Assessment of Wild Mushrooms from the Western Ghats, India: An in Vitro and Sub-Acute in Vivo Study
Source: Front Pharmacol. 2018 Feb 13;9:90. doi: 10.3389/fphar.2018.00090 (PMC5816808; doi:10.3389/fphar.2018.00090)
Supplement: Supplementary file 8 [file Table8.DOCX]

| **SL.NO** | **RT** | **NAME** | **IUPAC NAME** | **MOL.WT**  **(g/mol)** | **MOL. FORMULA** | **STRUCTURE** | **Reference number** |
| --- | --- | --- | --- | --- | --- | --- | --- |
| 1. | 14.13 | Prenyl alcohol | **3-Methyl-2-buten-1-ol** | 86.134 | [C_5_H_10_O](https://pubchem.ncbi.nlm.nih.gov/search/#collection=compounds&query_type=mf&query=C5H10O&sort=mw&sort_dir=asc) | 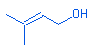 | NIST CAS # 556-82-1 #ions=51 |
| 2. | 15.95 | 1-propylpiperidine | 1-Propylpiperidine | 127.231 | C_8_H_7_N | 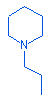 | NIST CAS # 5470-02-0 #ions=75 |
| 3. | 17.1 | Tridecanoic acid, methyl ester | Methyl tridecanoate | 228.376 | C_14_H_28_O_2_ | 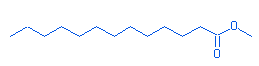 | NIST MS 1 OF 100  (1731-88-0) #ions=68 |
| 4. | 17.9 | Estradiol, 3-deoxy | **(17β)-Estra-1,3,5(10)-trien-17-ol** | 256.383 | C_18_H_24_O | 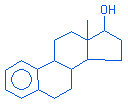 | NIST MS 4 OF 100  (2529-64-8) #ions=168 |
| 5. | 18.8 | 11-Octadecenoic acid, methyl ester,(Z) | **Methyl (12E)-12-octadecenoate** | 296.495 | C_19_H_36_O_2_ | 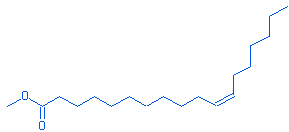 | NIST MS 1 OF 100 (1937-63-9 #ions=233 |
| 6. | 19.03 | Octadecanoic acid, methyl ester | Methyl stearate | 298.504 | C_19_H_38_O_2_ | 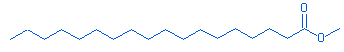 | NIST MS 3 OF 100  (112-61-8) #ions=107 |
| 7. | 19.68 | Oleic acid | **(9Z)-9-Octadecenoic acid** | 282.468 | C_18_H_34_O_2_ | 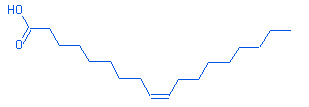 | NIST MS 1 OF 100  (112-80-1) #ions=247 |
| 8. | 20.88 | Eicosanoic acid, methyl ester | Methyl icosanoate | 326.565 | C_21_H_42_O_2_ | 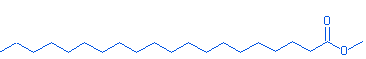 | NIST MS 5 OF 100  (1120-28-1) #ions=151 |
| 9. | 22.25 | 18,19-Secoyohimban-19-oic acid 16,17,20,21-tetrahydro-16-(hydroxymethyl), methyl ester,(15a’,16E)- | - | 352.00 | C_21_H_24_O_3_N_2_ | 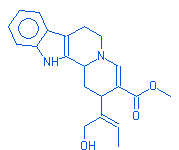 | NIST MS 6 OF 100  (5523-49-9) #ions=246 |
| 10. | 22.92 | Docosanoic acid, methyl ester | Methyl docosanoate | 354.619 | C_23_H_46_O_2_ | 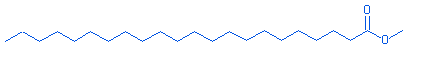 | NIST MS 1 OF 100  (929-77-1) #ions=191 |
| 11. | 25.83 | 9-octadecenoic acid (Z)-, 2-butoxyethyl ester | **2-Butoxyethyl (9E)-9-octadecenoate** | 382.620 | C_24_H_46_O_3_ | 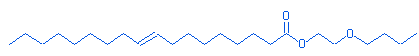 | NIST MS 28 OF 100  (109-39-7) #ions=230 |

**Table 8-Compounds present in *Psilocybe subcubenis* (PS) extract analysed using GC-MS**
