# Supplementary material for: Toxicity Assessment of Wild Mushrooms from the Western Ghats, India: An in Vitro and Sub-Acute in Vivo Study
Source: Front Pharmacol. 2018 Feb 13;9:90. doi: 10.3389/fphar.2018.00090 (PMC5816808; doi:10.3389/fphar.2018.00090)
Supplement: Supplementary file 9 [file Table9.DOCX]

| **Sl.No.** | **Mushroom species**  **Under the study** | **Occurrence** | **References** |
| --- | --- | --- | --- |
| 1. | *Agaricus endoxanthus* | Sri lanka^1^, Pakistan^2^, Spain^3^, Thailand^4^, Budapest^5^, Switzerland^5^, Malaysia^5^, Hawaii^6^ | 1. Pethiyagoda, R. The Family de Alwis Seneviratne of Sri Lanka: pioneers in biological illustration. 2. Ahmad, S., Iqbal, S. H., & Khalid, A. N. (1997). *Fungi of Pakistan*. SH Iqbal. 3. Parra LA, Villarreal M, Esteve-Ravento´ s F. 2002. Agaricus endoxanthus una specie tropicale trovata in Spagna. Rivista Micol 3:225–233. 4. Zhao, R. L., Desjardin, D. E., Callac, P., Parra, L. A., Guinberteau, J., Soytong, K., ... & Hyde, K. D. (2013). Two species of Agaricus sect. Xanthodermatei from Thailand. *Mycotaxon*, *122*(1), 187-195. 5. Chen, J., Parra, L. A., De Kesel, A., Khalid, A. N., Qasim, T., Ashraf, A., ... & Callac, P. (2016). Inter-and intra-specific diversity in Agaricus endoxanthus and allied species reveals a new taxon, A. punjabensis. *Phytotaxa*, *252*(1), 1-16. 6. Zhou, J. L., Su, S. Y., Su, H. Y., Wang, B., Callac, P., Guinberteau, J., ... & Zhao, R. L. (2016). A description of eleven new species of Agaricus sections Xanthodermatei and Hondenses collected from Tibet and the surrounding areas. Phytotaxa, 257(2), 99-121. |
| 2. | *Amanita angustilamellata* | Thailand^7,8,9^, China ^10,11,12^ | 1. Høiland, K., & Schumacher, T. (1982). Agarics, clavarioid and some heterobasidiomycetous fungi from northern Thailand. *Nordic Journal of Botany*, *2*(3), 265-271. 2. Saengwanit, U. (1992). Studies on Ectomycorrhizae of Dipterocarpus alatus. *[Anusan Mai-at Bangna (Thailand)]*. 3. Sanmee, R., Tulloss, R. E., Lumyong, P., Dell, B., & Lumyong, S. (2008). Studies on Amanita (Basidiomycetes: Amanitaceae) in Northern Thailand. *Fungal Divers*, *32*, 97-123. 4. Yang, Z. L. (1997). Die Amanita-Arten von Südwestchina. 5. Weiß, M., Yang, Z. L., & Oberwinkler, F. (1998). Molecular phylogenetic studies in the genus Amanita. *Canadian Journal of Botany*, *76*(7), 1170-1179. 6. Tang, L. P., Cai, Q., Lee, S. S., Buyck, B., Zhang, P., & Yang, Z. L. (2015). Taxonomy and phylogenetic position of species of Amanita sect. Vaginatae sl from tropical Africa. *Mycological progress*, *14*(6), 39 |

**Table 9:**

| 3. | *Chlorophyllum molybdites* | Israel^13^, Hawaii^14^, San Diego^15^, California^16^, Midwest United States^17^, Australia^18^, Florida ^19^, Argentina^20^, Japan^21, 22^, Brazil^23^, Burkina Faso (West Africa) ^24^ | 1. Avizohar. Z. (1967). Chlorophyllum molybdites (meyer ex fr) mass in Israel. *Israel journal of botany*, *16*(2), 63. 2. Smith, C. W. (1980). Mushroom poisoning by Chlorophyllum molybdites in Hawaii. *Hawaii medical journal*, *39*(1), 13-14. 3. Blayney, D. O. U. G. L. A. S., Rosenkranz, E. L. I. O. T., & Zettner, A. L. F. R. E. D. (1980). Mushroom poisoning from Chlorophyllum molybdites. *Western Journal of Medicine*, *132*(1), 74. 4. Levitan, D., Macy, J. I., & Weissman, J. (1981). Mechanism of gastrointestinal hemorrhage in a case of mushroom poisoning by Chlorophyllum molybdites. *Toxicon*, *19*(1), 179-180. 5. Lehmann, P. F., & Khazan, U. (1992). Mushroom poisoning by Chlorophyllum molybdites in the Midwest United States. *Mycopathologia*, *118*(1), 3-13. 6. Young, T. (1989). Poisonings by Chlorophyllum molybdites in Australia. *Mycologist*, *3*(1), 11-12. 7. Stenklyft, P. H., & Lynn Augenstein, W. (1990). Chlorophyllum molybdites-severe mushroom poisoning in a child. *Journal of Toxicology: Clinical Toxicology*, *28*(2), 159-168. 8. Soto, M. K., Carmarán, C. C., & Bruzzi, H. (2000). Suspected poisoning of domestic dogs by Macrolepiota molybdites. *Mycologist*, *14*(2), 50-51. 9. Ohta, T., Inoue, H., Kusano, G., & Oshima, Y. (1998). Lepiotins A and B, new alkaloids from the mushrooms, Macrolepiota neomastoidea and Chlorophyllum molybdites. *Heterocycles*, *2*(47), 883-891. 10. Kobayashi, Y., Kobayashi, K., Umehara, K., Dohra, H., Murata, T., Usui, T., & Kawagishi, H. (2004). Purification, characterization, and sugar binding specificity of an N-glycolylneuraminic acid-specific lectin from the mushroom Chlorophyllum molybdites. *Journal of Biological Chemistry*, *279*(51), 53048-53055. 11. Amazonas, M. A. L. D. A., Rubio, G. B. G., & Curial, R. M. (2007). Incidences of poisonings due to Chlorophyllum molybdites in the state of Paraná, Brazil. *Tecpar*. 12. Guissou, K. M. L., Yorou, N. S., Sankara, P., & Guinko, S. (2015). Assessing the toxicity level of some useful mushrooms of Burkina Faso (West Africa). *Journal of Applied Biosciences*, *85*(1), 7784-7793. |
| --- | --- | --- | --- |

| 4. | *Clarkeinda trachodes* | China^25^, Italy^26^, Africa^27^, Nothern Thailand^28^, Bangladesh^29^, Sri Lanka^30^, Malyasia^31^ | 1. Zhu-Liang, Y. A. N. G. (1991). Clarkeinda trachodes, an agaric new to China. *Acta botanica yunnanica*, *13*(3), 279-282. 2. Carmine & M. Contu (2002). *Clarkeinda trachodes*, one new species for the micoflora Italian recovered in Calabria. *Bulletin of group micologico New G. bresadola Series* 45(1): 33–39. 3. Zhao, R., Karunarathna, S., Raspé, O., Parra, L. A., Guinberteau, J., Moinard, M., ... & Guelly, A. K. (2011). Major clades in tropical Agaricus. *Fungal Diversity*, *51*(1), 279-296. 4. Clarkeinda trachodes, collection ecv3838 Vellinga, E. C., Sysouphanthong, P., & Hyde, K. D. (2011). The family Agaricaceae: phylogenies and two new white-spored genera. *Mycologia*, *103*(3), 494-509. 5. Hosen, I., & Ge, Z. W. (2012). Clarkeinda trachodes (Agaricales, Basidiomycetes), first record from Bangladesh. *Mycotaxon*, *118*(1), 331-336. 6. Karunarathna, S. C., Udayanga, D., Maharachchikumbura, S. N., Pilkington, M., Manamgoda, D. S., Wijayawardene, D. N. N., ... & Hyde, K. D. (2012). Current status of knowledge of Sri Lankan mycota. *Current Research in Environmental & Applied Mycology*, *2*(1), 18-29. 7. Pegler, D.N. (1986). *Agaric Flora of Sri Lanka*. Kew Bulletin of Additional Series–12, 328pp. |
| --- | --- | --- | --- |
| 5. | *Psilocybe subcubensis* | Japan^32, 33^, Nepal^34^, Mexico^35^,Congo^36^ | 1. Keller T, Schneider A, Regenscheit P, Dirnhofer R, Rücker T, Jaspers J, Kisser W (1999) Analysis of psilocybin and psilocin in *Psilocybe subcubensis* GUZMÁN by ion mobility spectrometry and gas chromatography–mass spectrometry.Forensic Sci Int 99:93–105 2. Gonmori, K., & Yoshioka, N. (2003). The examination of mushroom poisonings at Akita University. *Legal Medicine*, *5*, S83-S86. 3. Guzmán, G., & Kasuya, T. (2004). The known species of Psilocybe (Basidiomycotina, Agaricales, Strophariaceae) in Nepal. *Mycoscience*, *45*(4), 295-297. 4. Guzmán, G. (2008). Hallucinogenic mushrooms in Mexico: An overview. *Economic Botany*, *62*(3), 404-412. 5. Guzmfml, G., Nixon, S. C., Ramirez-Guillen, F., & Cortes-Perez, A.(2014). Psilocybe s. str.(Agaricales, Strophariaceae) in Africa with description of a new species from the Congo. *Sydowia*, *66*(1), 43-53. |
