# Supplementary figures and images for: Toxicity Assessment of Wild Mushrooms from the Western Ghats, India: An in Vitro and Sub-Acute in Vivo Study
Source: Front Pharmacol. 2018 Feb 13;9:90. doi: 10.3389/fphar.2018.00090 (PMC5816808; doi:10.3389/fphar.2018.00090)

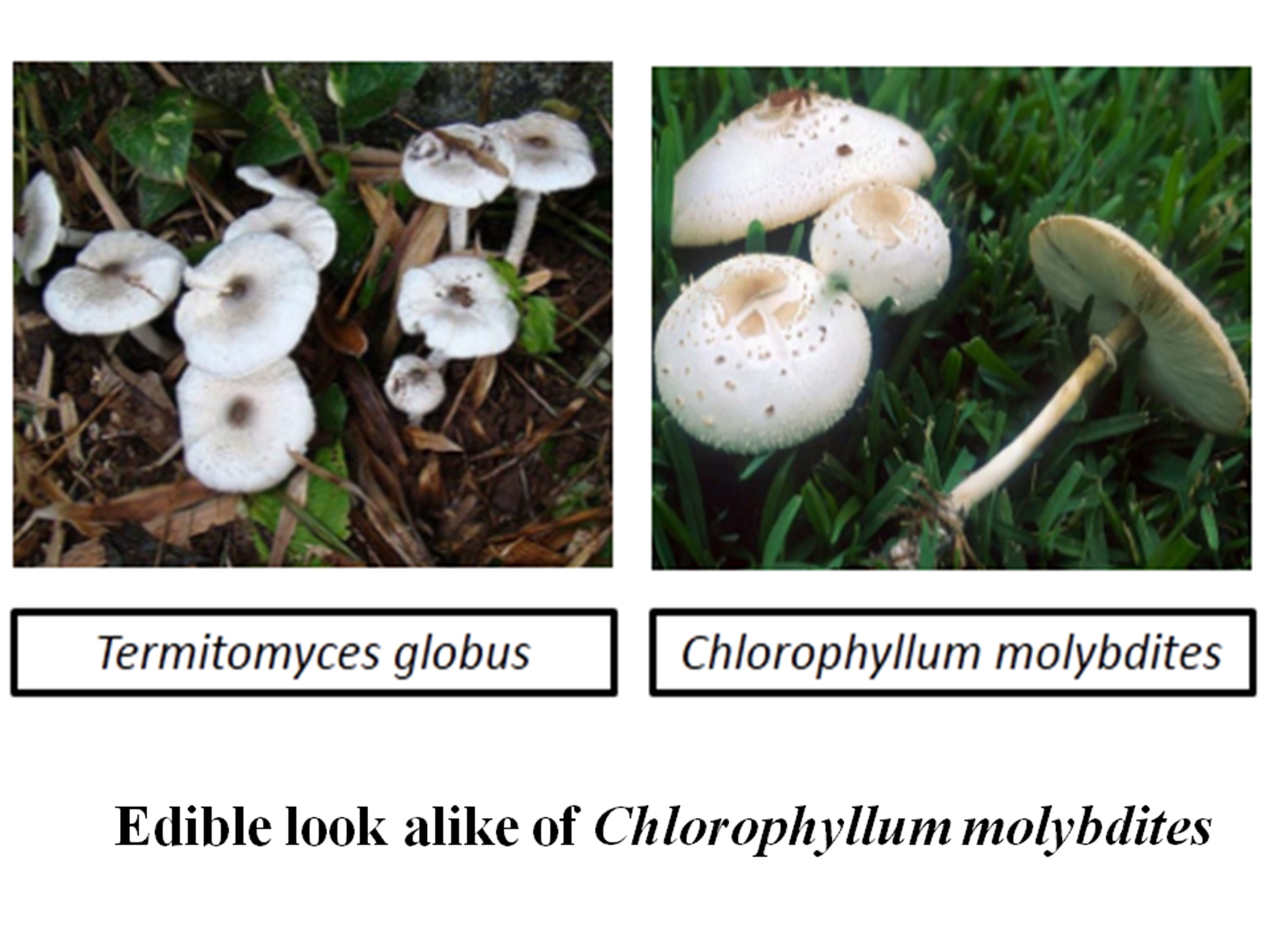

Supplement: Supplementary file 10 [file Image1.TIF]

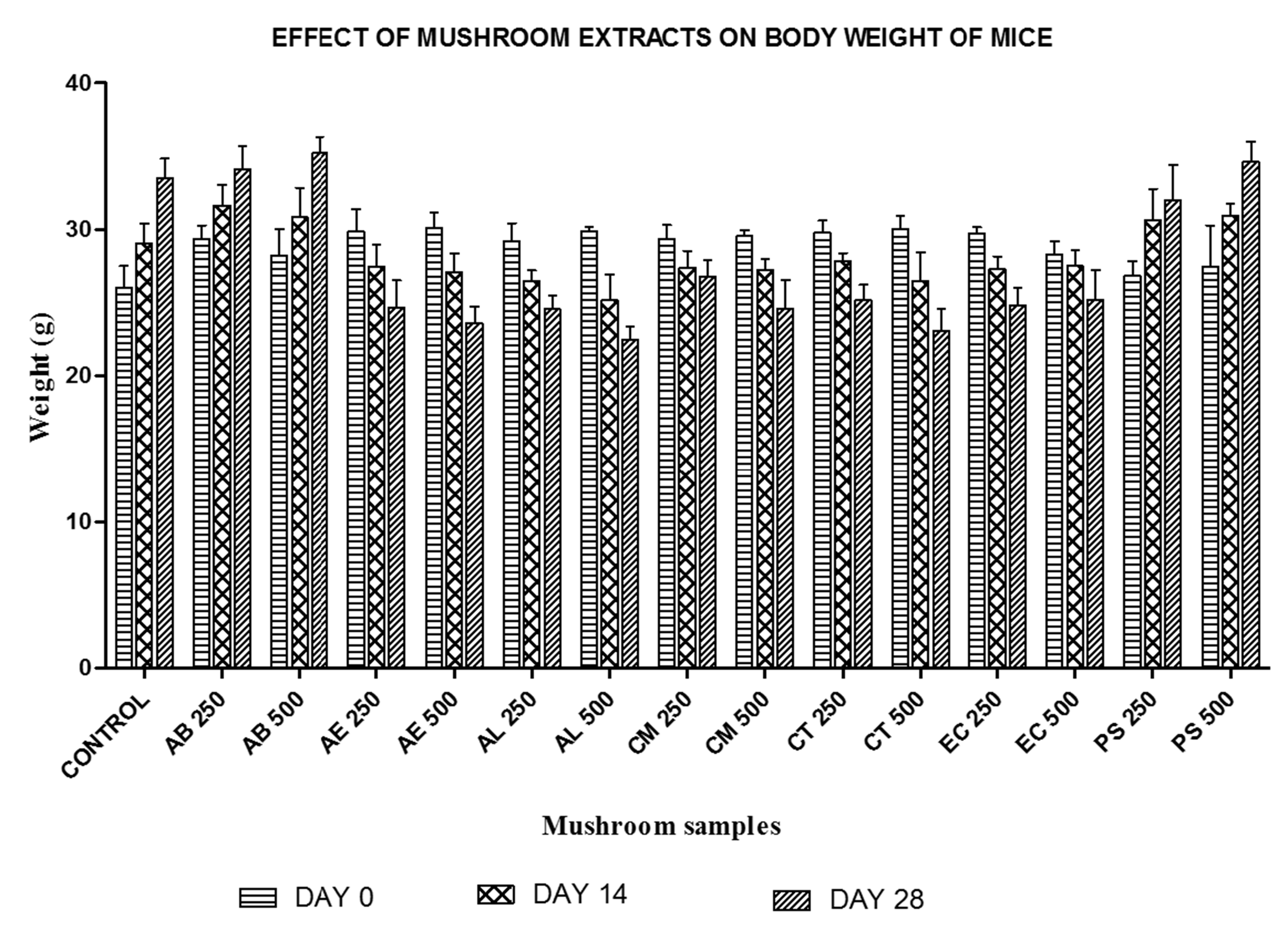

Supplement: Supplementary file 11 [file Image2.TIF]
